# Intratumoral α-SMA Enhances the Prognostic Potency of CD34 Associated with Maintenance of Microvessel Integrity in Hepatocellular Carcinoma and Pancreatic Cancer

**DOI:** 10.1371/journal.pone.0071189

**Published:** 2013-08-05

**Authors:** Wen-Quan Wang, Liang Liu, Hua-Xiang Xu, Guo-Pei Luo, Tao Chen, Chun-Tao Wu, Yong-Feng Xu, Jin Xu, Chen Liu, Bo Zhang, Jiang Long, Zhao-You Tang, Xian-Jun Yu

**Affiliations:** 1 Department of Pancreatic and Hepatobiliary Surgery, Fudan University Shanghai Cancer Center; Department of Oncology, Shanghai Medical College, Fudan University; and Pancreatic Cancer Institute, Fudan University, Shanghai, China; 2 Liver Cancer Institute, Zhongshan Hospital, Fudan University, Key Laboratory for Carcinogenesis & Cancer Invasion, Chinese Ministry of Education, Shanghai, China; Medical University Graz, Austria

## Abstract

Microvessel density (MVD) as an angiogenesis predictor is inefficient per se in cancer prognosis. We evaluated prognostic values of combining intratumoral alpha-smooth muscle actin (α-SMA)-positive stromal cell density and MVD after curative resection in hypervascular hepatocellular carcinoma (HCC) and hypovascular pancreatic cancer (PC). Tissue microarrays were constructed from tumors of 305 HCC and 57 PC patients who underwent curative resection and analyzed for α-SMA and CD34 expression by immunostaining. Prognostic values of these two proteins and other clinicopathological features were examined. Both low α-SMA density and high MVD-CD34 were associated in HCC with the presence of intrahepatic metastasis and microvascular invasion, and they were related to lymph node involvement and microvascular invasion in PC (*p*<0.05). Although CD34 alone, but not α-SMA, was an independent prognostic factor for overall survival and recurrence-free survival, the combination of low α-SMA and high CD34 was a predictor of worst prognosis for both types of tumors and had a better power to predict patient death and early recurrence (*p*<0.01). Furthermore, the results show that distribution of most of the α-SMA-positive cells and vascular endothelial cells overlap, showing major colocalization on vascular walls. Poor microvessel integrity, as indicated by high MVD, together with low perivascular α-SMA-positive cell coverage is associated with early recurrence, unfavorable metastasis, and short survival after tumor resection. This finding highlights the significance of vascular quality in tumor progression, which provides an optimized complement to vascular quantity in prognosis of postoperative patients.

## Introduction

Dysregulation of angiogenesis is indispensable for tumor metastasis, and it is one of the hallmarks of cancer [1]. Neovascularization supplies not only oxygen and nutrients to proliferative tumor cells but serves also as the conduit for migration [2]. Microvessel density (MVD) is the most recognized indicator to evaluate angiogenesis of solid tumors. Immunostaining of a vascular endothelial cell (EC) marker, such as CD34, is used to label MVD [3]–[6]. It has been reported that MVD is an adverse predictor in several cancers [7], [8], including hepatocellular carcinoma (HCC) [3], [4] and pancreatic cancer (PC) [9]. However, paradoxical results have also been noted [10], [11].

The tumor microenvironment plays an essential role in tumorigenesis and progression. Aside from vascular EC, carcinoma-associated fibroblast (CAF) is the major cell component in this milieu [12], [13]. It remains controversial whether stromal cells that are immunopositive for alpha-smooth muscle actin (α-SMA) represent activated CAF in intra/peritumoral tissues [14], [15]. Although we previously reported that peritumoral α-SMA-positive cells correlate with poor outcome of patients with HCC [16], the prognostic potency of intratumoral α-SMA is an open question. We recently observed colocalization of α-SMA- and CD34-positive staining in both intra- and peritumoral tissue, and we believe the significance of the finding is worth exploration.

Recently, some clinical studies have revealed that the success of antiangiogenic monotherapy is generally unimpressive, with low objective response rates and non-meaningful survival benefits [17]–[19]. Other preclinical studies have shown that antiangiogenesis inhibits tumor growth but accelerates metastasis [20], [21]. These findings were probably related to a focus on vessel quantity alone, while neglecting the quality of vasculature, namely, microvessel integrity (MVI). In other words, antiangiogenesis might reduce MVD but impair MVI, thus leading to metastasis [22]. Actually, the tumor vascular wall is composed of a continuum of cell types, ranging from EC, vascular smooth muscle cells, and pericytes [23], of which the latter two are classified as perivascular cells (PVCs) [19]. Given that MVD is a marker only for EC, it cannot represent the integral vasculature. More importantly, we have found a colocalization pattern for α-SMA and CD34, as well as additional EC markers described by other groups [19], [24]–[26]. On this basis, we hypothesized that the concerted function of perivascular α-SMA-positive cells and ECs in the vascular wall is to stabilize tumor vessels and block tumor cell migration via intra/extravasation. Therefore, a combination of α-SMA and CD34 as predictive markers (CD34^+^/α-SMA^+^) might be useful for evaluation of MVI. Much of the accumulating evidence about MVI in cancer comes from preclinical studies. Its significance in the clinic, however, is unknown.

Two common and lethal malignancies, which contrast in terms of intra/peritumoral vasculature, are HCC and PC. Tissues of HCC are highly vascularized with lower stromal content [3], whereas those of PC are poorly vascularized but with abundant stroma [13]. In this study, we carried out vascular integrity analysis of these two tumor types, having significant representation. We investigated the prognostic value of α-SMA separately and combinatorially with MVD after curative resection of the primary tumor, initially in HCC and then in PC. Results in PC confirm those found in HCC.

## Materials and Methods

### Patients, Specimens, Follow-Up, and Postoperative Treatment

A total of 305 patients (Cohort 1, Table S1 in in File S1) who underwent curative liver resection for pathology-proven HCC at the Liver Cancer Institute of Zhongshan Hospital, Fudan University, were examined. None of them received any preoperative anticancer treatment. These patients were observed between October 2004 and November 2010, with a total follow-up time of 72 months. The criteria for resectability, collection of specimens, determination of tumor stage and differentiation, and follow-up procedures have been described elsewhere [27]–[29]. Overall survival (OS) and recurrence-free survival (RFS) were defined as the interval between dates of surgery and death, and between dates of surgery and recurrence, respectively. If recurrence was suspected, computerized tomography scanning or magnetic resonance imaging was performed immediately; if recurrence had not been diagnosed, patients were observed until death or the last follow up. At the last follow up of the study, 132 patients had tumor recurrence, and 108 were found to have died. The 1-, 3-, and 5-year OS rates were 88%, 65%, and 64%, respectively; the recurrence rates over those same time intervals were 25%, 42%, and 43%, respectively.

From January 2010 to June 2011, 179 consecutive patients with pathology-proven PC underwent curative resection at our institute, with surgery performed by the same team [30]. The resectable criteria met the National Comprehensive Cancer Network (NCCN) Clinical Practice Guidelines in Oncology-Pancreatic Cancer Guideline 2010 (http://www.nccn.org). Of the PC patients, 57 cases (Cohort 2, Table S2 in File S1) randomly retrieved from a prospectively collected database were identified as having no microscopically observable residual tumor (R0). None of them received any preoperative anticancer treatment. Entire tumors were collected. Tumors were staged according to the tumor-node-metastasis (TNM) classification system [31]. Tumor differentiation was graded by the NCCN Guideline. Lymph node involvement was determined from postoperative pathological diagnosis. All patients were monitored until July 2012, with a median follow-up time of 15.5 months. Treatment modalities after relapse were administered also according to the NCCN Guideline. The rates of OS and RFS were defined as above. At the last follow up, 33 patients had tumor recurrence, and 22 had died. The 0.5-, 1-, and 2-year OS rates were 98%, 86%, and 61%, respectively; and the 0.5-, 1-, and 2-year recurrence rates over the same time intervals were 21%, 42%, and 58%, respectively. Another independent test cohort 3, including 52 PC patients treated at our institute, was also collected for the study (Table S3 in File S1).

This study was approved by the research ethics committees of Zhongshan Hospital and the Fudan University Shanghai Cancer Center. The written informed consent was obtained from each patient before participating in this study according to the two committees’ regulations.

### Tissue Microarray Construction, Immunohistochemistry, and Evaluation

Tissue microarray (TMA) construction was as described [28]. Briefly, two cores for cohort 1 and three cores for cohorts 2 and 3, drilled from each representative formalin-fixed, paraffin-embedded tumor tissue, were used to make TMA slides (Shanghai Biochip Company Ltd, Shanghai, China). Two or three cylinders from different areas of tumor samples were obtained; accordingly, a total of four TMA chips for cohort 1 and two chips for cohorts 2 and 3 were prepared.

The marker used for PVC was α-SMA [13], [19], and CD34 was used as marker for EC [3], [4]. Hypoxia-inducible factor 1α (HIF-1α) and carbonic anhydrase IX (CA IX) were selected as the tumor hypoxia biomarker [24], [32], [33]. Immunohistochemical (IHC) analysis of these markers in TMA sections (4-µm thick) was done by a two-step method as described [29]. The primary rabbit monoclonal anti-human α-SMA antibody (1∶100; Abcam, Cambridge, MA), mouse monoclonal anti-human CD34 (1∶100; Abcam), rabbit monoclonal anti-human CA IX (1∶150; Abcam), and mouse monoclonal anti-human HIF-1α (1∶100; Sigma, St. Louis, MO) were used. Incubation with primary antibodies was performed overnight at 4°C in a wet chamber. The Envision-plus detection system with an anti-rabbit/mouse polymer (EnVision +/HRP/Mo, Dako, Glostrup, Denmark) was employed. Reaction products were visualized by incubation with 3,3′-diaminobenzidine. Negative controls were treated identically but with omission of primary antibody.

Density of positive staining in whole view was measured using a computerized image system composed of a Leica charge-coupled device camera, DFC500, connected to a Leica DM IRE2 microscope (Leica, Cambridge, UK). Images of five representative fields at ×200 magnification were captured by the Leica QWin Plus v3 software. An identical setting was used for all images. For evaluation of vascular density, vascular integrity, and hypoxia intensity, area and integrated optical density (IOD) of immunostaining in each image were measured using Image-Pro Plus v6.2 software [28]. Results were quantified as α-SMA- or CD34-positive area/total area, and HIF-1α or CA IX IOD/total area.

### Immunohistochemical Staining of Serial Sections

Fifteen pairs (total thirty) of tumorous and matched peritumoral samples (tissue adjacent to the tumor within a distance of 10 mm) from patients with HCC and PC were collected and used for preparing serial paraffin-embedded and frozen tissue slides. The paraffin section slides were used for IHC of α-SMA and CD34. Eight cross-sections from each sample were subjected to qualitative analysis. The IHC procedure was described earlier [29]. Under ×200 magnification, images of representative fields in the same location of paired serial sections with positive α-SMA and CD34 staining were captured using a computerized image system.

### Immunofluorescent Double Staining for α-SMA and CD34

Frozen sections were used for immunofluorescent double staining of α-SMA and CD34. The protocol was as described [29]. After reaction with the primary antibodies (1∶80) and subsequent rinsing, sections were incubated with both Cy3-conjugated goat anti-rabbit and fluorescein isothiocyanate-conjugated goat anti-mouse antibodies (both 1∶100; Jackson, West Grove, PA) and counterstained with 4′,6-diamidine-2′-phenylindole dihydrochloride (DAPI) to stain nuclei. Representative images were acquired by laser confocal microscopy.

### Statistical Analysis

All statistical analyses were performed with SPSS 16.0 software. The Pearson chi-square or Fisher’s exact test was used to compare qualitative variables, and quantitative variables were analyzed by *t-*test or Spearman test. The cutoff point of α-SMA or CD34 density for definition of subgroups was the median value (Fig. S1 in File S1). The clinicopathological features were compared between the two risk groups using a Mann-Whitney test. For survival analysis, Kaplan-Meier curves were drawn, and differences between the curves were calculated by Log-rank test. Independent prognostic significance of risk factors identified by univariate analysis was computed by the Cox proportional hazards model. Receiver operating characteristic (ROC) curve analysis was used to determine the predictive value among parameters. A value of *P*<0.05 was considered statistically significant.

## Results

### Patterns of Perivascular α-SMA-positive Stromal Cells and ECs Distribution

**Figure 1:**
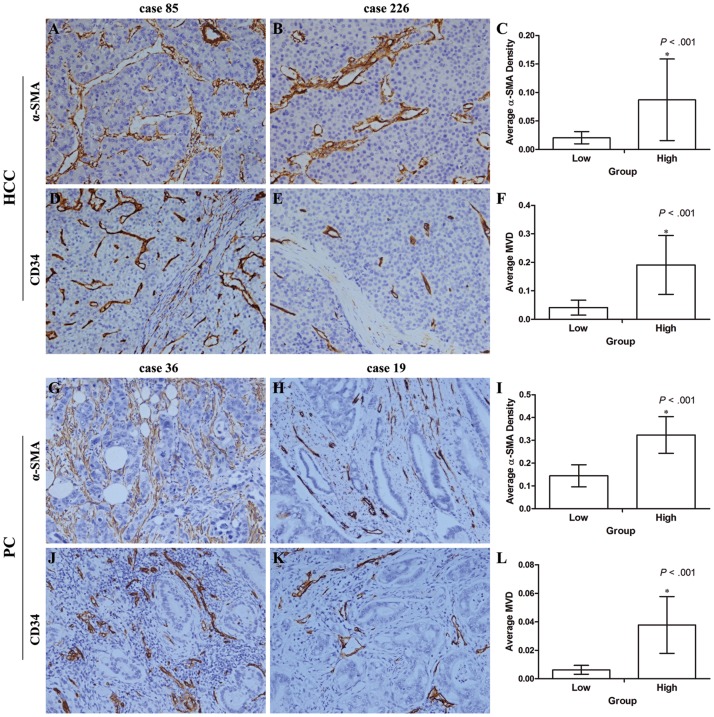
Representative high and low perivascular stromal cell densities and microvessel density (MVD) measured by immunostaining for alpha-smooth muscle actin (α-SMA) and CD34 in tissue microarrays of hepatocellular carcinoma (HCC) and pancreatic cancer (PC). Case 85 (HCC) and PC case 36 showed high α-SMA density (A, G) and MVD-CD34 values (D, J); whereas, HCC case 226 and PC case 19 showed low α-SMA density (B, H) and MVD (E, K) (×200). (C, F, I, L) Average α-SMA density and MVD of high or low risk groups in HCC and PC. ^*^Independent samples *t* test showed a statistical difference between the two groups.

**Figure 2:**
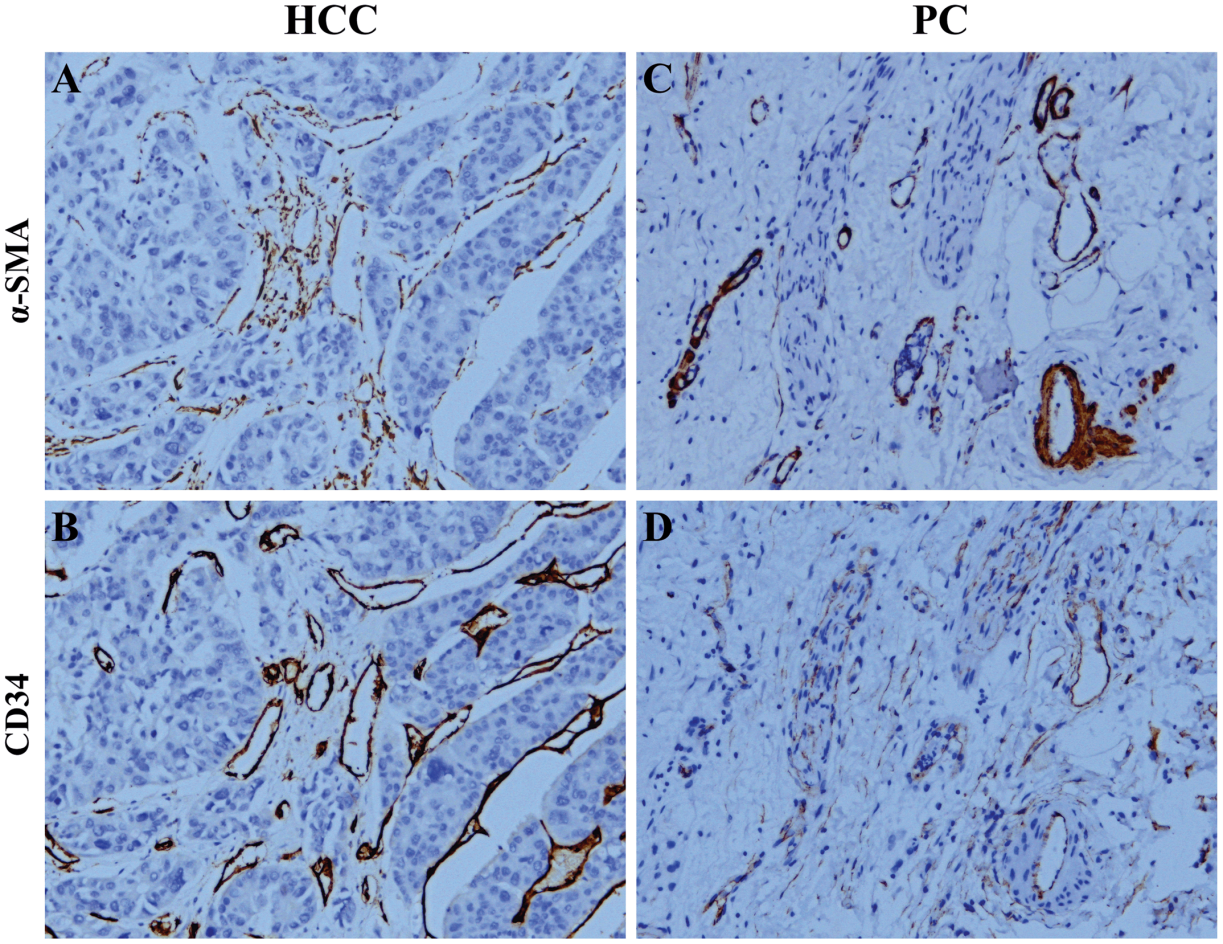
Distribution of perivascular stromal cells and endothelial cells (ECs) on tumor vascular walls, measured by alpha-smooth muscle actin (α-SMA) and CD34 as markers for immunostaining in hepatocellular carcinoma (HCC) and pancreatic cancer (PC) tissues. (Paired A and B, C and D) Immunohistochemical staining of serial sections showed a coexpression pattern of α-SMA and CD34 (×200).

In all tumors collected, HCC always showed a very low stromal content, while PC contained abundant stroma (Fig. 1). Representative high and low α-SMA/CD34 staining may be seen in this figure. The mean α-SMA-positive cell density for HCC was 0.0540±0.0611 (median of 0.0389; range, 0.000320 to 0.563), and it was 0.232±0.111 for PC (median, 0.202; range, 0.0494 to 0.474) (Fig. S1A and 1C in File S1). Specific staining of capillary-like vessels by anti-CD34 was observed in HCC (mean MVD-CD34 of 0.116±0.106; median, 0.0877; range, 0.00102 to 0.545) and PC (mean MVD, 0.0217±0.0212; median, 0.0112; range, 0.00103 to 0.0928) (Fig. S1B and 1D in File S1). A rich content of microvessels was found in HCC, while the content of microvessels in PC was poor (Fig. 1). Compared with the homogeneous vessel pattern in peritumoral normal liver or pancreas tissue, the intratumoral vascular morphology was heterogeneous (Fig. 1; Fig. S2 in File S1). Immunohistochemistry of serial sections revealed a colocalization of α-SMA and CD34 staining distributions (Fig. 2A and 2B, 2C and 2D), and a similar appearance was also found in peritumoral tissue (Fig. S3 in File S1). This phenomenon was confirmed by immunofluorescent double staining of α-SMA and CD34, where α-SMA-positive cells were seen to wrap around EC on vascular walls (Fig. S4 in File S1).

### Correlations between Tumor α-SMA-positive Cell Density or MVD and Clinicopathological Features

When the median value was set as the cutoff point for α-SMA and CD34 densities, patients were divided into subgroups of high or low risk (Fig. 1; Fig. S1 in File S1). As shown in Table 1 (detailed in Tables S4 and S5 in File S1), in HCC, patients with a low α-SMA density were prone to have presence of intrahepatic metastasis and microvascular invasion, and low tumor differentiation; high MVD was associated with large tumor size, high serum α-fetoprotein concentration, presence of intrahepatic metastasis and microvascular invasion, and high TNM stage. In PC, low α-SMA was related to high nodal involvement, microvascular invasion, low tumor differentiation, and high TNM stage; high MVD correlated with large tumor size, high lymph nodal involvement rate, and presence of microvascular invasion. In HCC patients with cirrhosis (stage 4; *n* = 72), there were significantly lower α-SMA values than in patients without cirrhosis (stages 1 to 3; *n* = 233; 18.30% vs. 28.95%; *p* = 0.029); MVD was equally distributed between the two subgroups (22.88% vs. 24.34%; *p* = 0.763).

**Table 1:** Relationship between tumor α-SMA-positive cell density and microvessel density and clinicopathological features.

Variables	α-SMA Density					MVD				
	High		Low		*P*	High		Low		*P*
	No. of Patients	%	No. of Patients	%		No. of Patients	%	No. of Patients	%	
HCC (Cohort 1)	n = 153		n = 152			n = 153		n = 152		
Tumor size, cm*	5.28±3.37		5.92±4.36		.151†	6.79±4.66		4.40±2.44		.000†
AFP, ng/dl*	5578.41±14991.00		5588.57±13849.30		.995	7667.41±17286.92		3485.82±10410.35		.011†
Hepatitis B history					.530					.108
Yes	129	84	132	87		126	82	135	89	
No	24	16	20	13		27	18	17	11	
Liver cirrhosis					.029					.763
Yes	28	18	44	29		35	23	37	24	
No	125	82	108	71		118	77	115	76	
Intrahepatic metastasis					.001					.007
Yes	12	8	33	22		31	20	14	9	
No	141	92	119	78		122	80	138	91	
Microvascular invasion					.018					.003
Yes	53	35	73	48		76	50	50	33	
No	100	65	79	52		77	50	102	67	
Tumor differentiation					.008					.066
Stage I–II	118	77	96	63		100	65	114	75	
Stage III–IV	35	23	56	37		53	35	38	25	
TNM stage					.074					.044
I	21	14	15	10		13	9	23	15	
II	55	36	74	49		60	39	69	45	
IIIA	77	50	63	41		80	52	60	40	
**PC (Cohort 2)**	**n = 29**		**n = 28**			**n = 28**		**n = 29**		
CA199, U/mL*	458.55±579.93		622.15±806.70		.385†	591.20±719.58		488.43±687.59		.584
Tumor size, group					.896					.024
≤3 cm (n = 28)	14	48	14	50		18	64	10	35	
>3 cm (n = 29)	15	52	14	50		10	36	19	65	
Nodal involvement					.046					.011
Yes	8	28	15	54		16	57	7	24	
No	21	72	13	46		12	43	22	76	
Microvascular invasion					.044‡					.044‡
Yes	3	10	9	32		9	32	3	10	
No	26	90	19	68		19	68	26	90	
Tumor differentiation					.042					.236
Grade 1–2	16	55	8	29		14	50	10	34	
Grade 3–4	13	45	20	71		14	50	19	66	
TNM stage					.024					.085
IB and IIA	21	72	12	43		13	46	20	69	
IIB	8	28	16	57		15	54	9	31	

* Mean ± standard deviation, Student’s *t*-test.

† Equal variances not assumed.

‡ Twenty-five percent of all the cells have expected count less than 5; Fisher’s exact test.

*p*<0.05 was considered statistically significant.

Abbreviations: α-SMA, alpha-smooth muscle actin; MVD, microvessel density; HCC, hepatocellular carcinoma; AFP, α-fetoprotein; TNM, tumor-node-metastasis; PC, pancreatic cancer; CA, carcinoembryonic antigen.

### Prognostic Impact of Tumor α-SMA-positive Cell Density or MVD on Postoperative Survival and Recurrence

**Figure 3:**
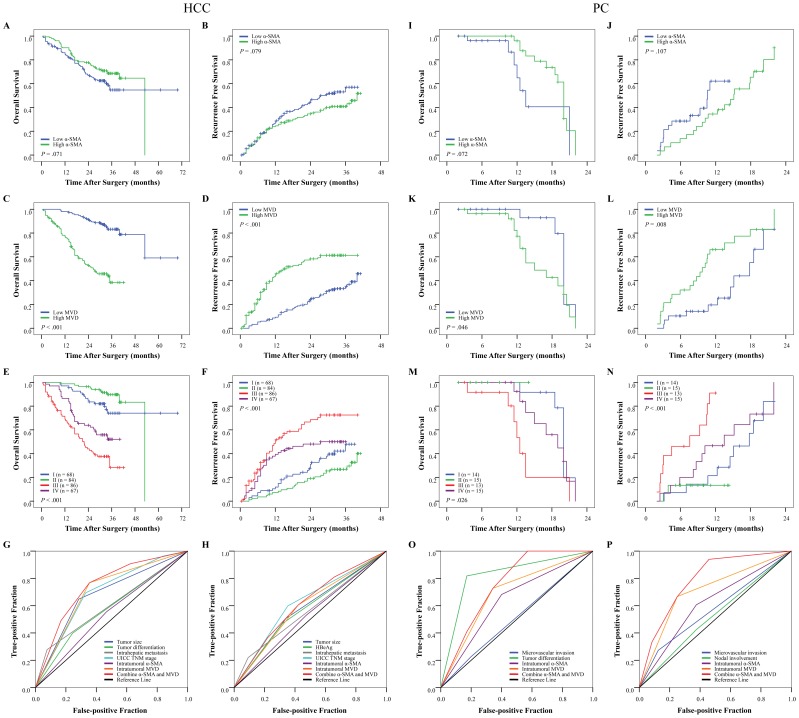
Cumulative overall survival (OS) and recurrence-free survival (RFS) curves of patients with low or high tumor alpha-smooth muscle actin (α-SMA) density, microvessel density (MVD), and their combination. (A, B, I, J) The α-SMA density was associated with neither OS nor RFS. (C, D, K, L) Low MVD was correlated with prolonged OS and RFS. (E, F, M, N) Patients in each cohort were classified into four groups according to their intratumoral α-SMA density and MVD. (G, H, O, P) The predictive values of marker combination and other risk factors identified by multivariate analysis were studied by receiver operating characteristic analysis (see Results for details). UICC, International Union against Cancer.

In univariate analysis of HCC, tumor size, tumor differentiation, presence of microvascular invasion and intrahepatic metastasis, and TNM stage were associated with both OS and RFS; positive hepatitis B e antigen was also associated with RFS. In PC, the presence of microvascular invasion was associated with both OS and RFS; tumor differentiation was also associated with OS; and nodal involvement was a potential impact factor of RFS (Table 2). The α-SMA values of both HCC and PC were not associated with OS or RFS (for HCC: *p* = 0.071 and *p* = 0.079, Fig. 3A and 3B; for PC: *p* = 0.072 and *p* = 0.107, Fig. 3I and 3J). The median OS and RFS times for patients with high MVD were 26.5 and 16.0 months for HCC, and 15.0 and 9.7 months for PC, respectively. These periods were significantly shorter than those for patients with low MVD (for HCC: 57.4 and 33.1 months, both *p*<0.001, Fig. 3C and 3D; for PC: 20.0 and 17.8 months, *p* = 0.046 and *p* = 0.008, Fig. 3K and 3L).

**Table 2:** Univariate and multivariate analysis of factors associated with survival and recurrence.

Factors	OS				RFS			
	Univariate *P*	Multivariate			Univariate *P*	Multivariate		
		HR	95% CI	*P*		HR	95% CI	*P*
HCC (Cohort 1)								
Tumor size: ≤5 vs.>5 cm	<.001	2.435	1.588–3.736	<.001	<.001	1.594	1.097–2.315	.014
HBeAg: negative vs. positive	.177			NA	.006	1.443	1.019–2.042	.039
Tumor differentiation: Stages I–II vs.III–IV	.001	1.661	1.112–2.482	.013	.011			NS
Microvascular invasion: no vs. yes	<.001			NS	.001			NS
Intrahepatic metastasis: no vs. yes	<.001	1.875	1.193–2.947	.006	<.001	2.055	1.320–3.200	.001
TNM stage: I vs. II vs. IIIA	<.001	0.368	0.128–1.063	.018	<.001	0.512	0.265–0.989	<.001
Intratumoral α-SMA density: low vs. high	.071			NS	.079			NS
Intratumoral MVD: low vs. high	<.001	4.236	2.659–6.747	<.001	<.001	2.585	1.784–3.745	<.001
Combine α-SMA density and MVD*,‡	<.001	1.381	0.830–2.296	<.001	<.001	1.325	0.801–2.191	<.001
PC (Cohort 2)								
Tumor size: ≤3 vs.>3 cm	.751			NA	.431			NA
Nodal involvement: no vs. yes	.724			NA	.077			NS
Microvascular invasion: no vs. yes	.018			NS	.001	3.267	1.375–7.764	.007
Tumor differentiation: Grades 1–2 vs. 3–4	.019	0.139	0.039–0.494	.002	.918			NA
TNM stage: IB and IIA vs.IIB	.250			NA	.389			NA
Intratumoral α-SMA density: low vs. high	.072			NS	.107			NS
Intratumoral MVD: low vs. high	.046	3.578	1.247–10.265	.018	.008	2.230	1.061–4.688	.034
Combine α-SMA density and MVD†,‡	.026	6.294	1.224–32.357	.028	<.001	2.534	0.866–7.411	.009

* Patients were classified into four groups according to their intratumoral α-SMA density and MVD: group I (n = 68), both low density; group II (n = 84), high α-SMA density and low MVD; group III (n = 86), low α-SMA density but high MVD; and group IV (n = 67), both high density.

† Group I (n = 14), both low density; group II (n = 15), high α-SMA density and low MVD; group III (n = 13), low α-SMA density but high MVD; and group IV (n = 15), both high density.

‡ The multivariate analysis of different subgroups of α-SMA and MVD was analyzed together with other risk factors identified by univariate analysis but excluding α-SMA and MVD themselves, in order to avoiding the interference of them on the combinational group.

Abbreviations: OS, overall survival; RFS, recurrence free survival; HR, hazard ratio; CI, confidence interval; HCC, hepatocellular carcinoma; HBeAg, hepatitis B e antigen; TNM, tumor-node-metastasis; α-SMA, alpha-smooth muscle actin; MVD, microvessel density; PC, pancreatic cancer; NA, not adapted; NS, not significant.

Risk factors identified by univariate analysis were pooled into a multivariate Cox proportional hazards analysis (Table 2; also detailed in Tables S6 and S7 in File S1). The results show that in both tumor types, α-SMA is not an independent risk factor of OS or RFS. High MVD was an independent risk factor of OS (for HCC: hazard ratio [HR] = 4.236, *p*<0.001; for PC: HR = 3.578, *p* = 0.018) and of RFS (for HCC: HR = 2.585, *p*<0.001; for PC: HR = 2.230, *p* = 0.034).

Taking into account the recurrence characteristics of HCC [34], we adopted 24 months as the cutoff value to separate early versus late subgroups of tumor recurrence. Unfortunately, no difference was found between patients with high and low α-SMA in either early recurrence (66 of 153 vs. 89 of 152 patients, *p* = 0.528; Fig. S5A in File S1) or late recurrence (87 of 153 vs. 63 of 152 patients, *p* = 0.665). More patients with high MVD (compared with patients with low MVD) had an early recurrence (110 of 153 vs. 45 of 152 patients, *p* = 0.001; Fig. S5B in File S1) rather than a late recurrence (43 of 153 vs. 107 of 152 patients, *p* = 0.429). For PC, 6 and 12 months were set as cutoff values for distinguishing early versus late recurrence [35], respectively; under these conditions, no significant difference was found for α-SMA and MVD between subgroups.

To eliminate the influence of tumor size on patient outcome, we further investigated the prognostic factors in the small-tumor HCC subgroup (maximum diameter of ≤5 cm, *n* = 179); in PC, tumor size did not correlate with OS or RFS. The MVD values were associated with OS and RFS (*p*<0.001 and *p* = 0.001, respectively) in this subgroup; whereas, α-SMA was related to neither OS nor RFS (*p* = 0.520 and *p* = 0.153, respectively; Fig. S6A to 6D in File S1). Detailed correlations of other factors with patient outcome for the small-tumor HCC subgroup are summarized in Table S8 in File S1.

### Prognostic Value of Combination of α-SMA-positive Cell Density and MVD and ROC Analysis

**Figure 4:**
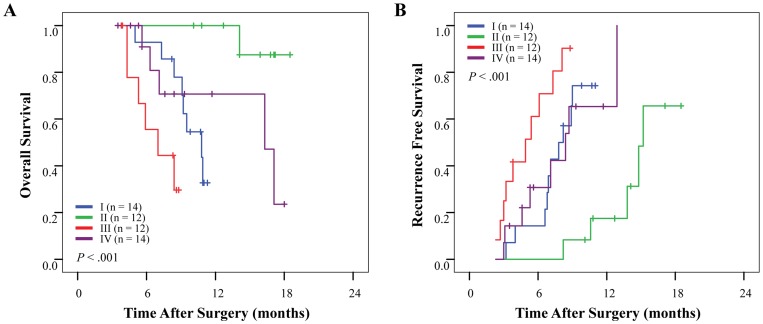
Cumulative (A) overall and (B) recurrence-free survival curves from the combination of tumor alpha-smooth muscle actin density and microvessel density in an independent test pancreatic cancer cohort 3.

Study patients were divided into four groups according to their intratumoral α-SMA density and MVD values: group I (HCC/PC: *n* = 68/14), low α-SMA and low MVD; group II (HCC/PC: *n* = 84/15), high α-SMA and low MVD; group III (HCC/PC: *n* = 86/13), low α-SMA and high MVD; and group IV (HCC/PC: *n* = 67/15), high α-SMA and high MVD (see Table S9 in File S1, the detailed clinicopathological features of patients of different subgroups of α-SMA and MVD). Prognostic analysis showed significant differences in OS rates (*p*<0.001 and *p* = 0.026 for HCC and PC, respectively) and RFS rates (*p*<0.001 for both) among the four combinational groups (Table 2). In HCC Cohort 1, the 5-year OS and RFS rates were 88.1% and 70.2%, respectively, for group II; but only 38.4% and 44.2%, respectively, for group III (Fig. 3E and 3F). In PC Cohort 2, the 2-year OS and RFS rates were 100% and 86.7%, respectively, for group II; but they were only 40.0% and 15.4%, respectively, for group III (Fig. 3M and 3N). The results of multivariate analysis showed that combination of α-SMA density and MVD was an independent prognostic factor for OS and RFS (Table 2 and Tables S6 and S7 in File S1). A similar result was found in early recurrence HCC (Fig. S5C in File S1) and small-tumor HCC subgroups (Fig. S6E and 6F, and Table S8 in File S1), and it was confirmed in the independent test PC Cohort 3 (Fig. 4).

Risk factors identified by multivariate analysis and the combination of α-SMA and MVD were adopted, and their predictive values were determined by ROC analysis (Table 3; detailed in Tables S10 and S11 in File S1). Tumor MVD predicted death and recurrence (*p*<0.05). Although α-SMA predicted neither death nor recurrence, the combination of α-SMA and MVD precisely predicted death and early recurrence (*p*<0.01 for all). Except for TNM stage and recurrence in HCC, and for tumor differentiation and death in PC, the predictive value of combination of α-SMA and MVD was greater than other factors. When the cutoff value was set as the group III (with low α-SMA and high MVD) versus other threes groups, the negative predictive value, positive predictive value, sensitivity, and specificity of HCC cohort 1 were 0.749, 0.616, 0.491, 0.832 for OS and 0.616, 0.558, 0.364, 0.780 for RFS, and of PC cohort 2 were 0.690, 0.600, 0.409, 0.829 for OS and 0.476, 0.733, 0.333, 0.833 for RFS, respectively. The areas under the curve of this combination were 0.743/0.758 (HCC/PC) for death (*p*<0.001 and *p* = 0.001, respectively) and 0.615/0.790 (HCC/PC) for recurrence (*p* = 0.001 and *p*<0.001, respectively) (Fig. 3G, 3H, 3O, and 3P).

**Table 3:** Prognostic values of variables for death and disease recurrence by receiver operating characteristic analysis.

Variables	Area undercurve	95% CI	*P* value
HCC (Cohort 1)			
Death			
Intratumoral α-SMA density	0.544	0.477–0.612	.201
Intratumoral MVD	0.707	0.646–0.767	.000
Combine α-SMA and MVD	0.743	0.686–0.800	.000
TNM stage	0.695	0.634–0.755	.000
2-year recurrence			
Intratumoral α-SMA density	0.528	0.463–0.594	.399
Intratumoral MVD	0.592	0.528–0.656	.006
Combine α-SMA and MVD	0.615	0.551–0.678	.001
TNM stage	0.621	0.558–0.685	.000
PC (Cohort 2)			
Death			
Intratumoral α-SMA density	0.641	0.493–0.789	.075
Intratumoral MVD	0.692	0.550–0.835	.015
Combine α-SMA and MVD	0.758	0.637–0.880	.001
Tumor differentiation	0.823	0.705–0.942	.000
2-year recurrence			
Intratumoral α-SMA density	0.616	0.467–0.764	.139
Intratumoral MVD	0.708	0.570–0.847	.008
Combine α-SMA and MVD	0.790	0.666–0.913	.000

Abbreviations: CI, confidence interval; HCC, hepatocellular carcinoma; α-SMA, alpha-smooth muscle actin; MVD, microvessel density; TNM, tumor-node-metastasis; PC, pancreatic cancer.

### Intensity of HIF-1α and CA IX Expression in Subgroups of Different MVI

**Figure 5:**
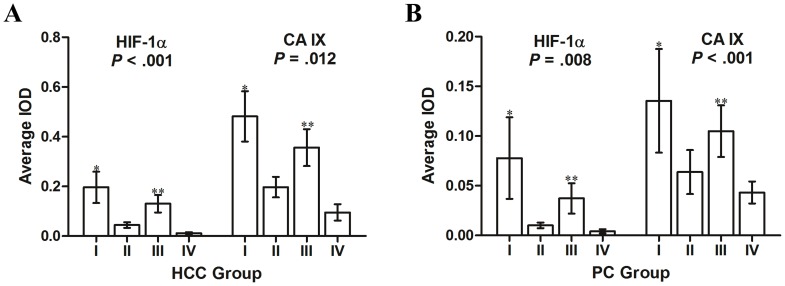
Intensity of hypoxia-inducible factor 1α (HIF-1α) or carbonic anhydrase IX (CA IX) expression in different patient subgroups by the combination of tumor alpha-smooth muscle actin density and microvessel density. The indicator of tumor hypoxia were HIF-1α and CA IX, and their intensities were divided into four groups, as described. The HIF-1α or CA IX density was greatest in group I and lowest in group IV for both (A) hepatocellular carcinoma (HCC) cohort 1, and (B) pancreatic cancer (PC) cohort 2; (*p*<0.001 and *p* = 0.008 for HIF-1α, and *p* = 0.012 and *p*<0.001 for CA IX, respectively). ^*^Compared with group I., and^**^compared with group III; both *p*<0.05. IOD, integrated optical density.

Values of HIF-1α and CA IX were used as the indicator of tumor hypoxia, and their intensities were divided into four groups according to the combination of α-SMA and MVD. The average HIF-1α or CA IX density was largest for combinational group I and smallest for group IV in HCC Cohort 1 and PC Cohort 2 (one-way analysis of variance, *p*<0.001 and *p* = 0.008 for HIF-1α, and *p* = 0.012 and *p*<0.001 for CA IX, respectively; Fig. 5). Compared with group I, the HIF-1α or CA IX of group II was significantly lower; compared with group III, HIF-1α or CA IX of group IV was also lower (both *p*<0.05).

## Discussion

In the present study, we have found that a low intratumoral α-SMA-positive cell density, together with high MVD-CD34 values, is significantly associated with a high incidence of intravascular tumor thrombus and poor survival after resection of HCC. Of these two markers, only MVD is an independent prognostic factor; however, the combination of the two has greater power to predict patient death and early recurrence. Furthermore, our work reveals that localization of most perivascular α-SMA-positive cells and ECs overlaps on vascular walls, and there is greater than 80% colocalization in the tumor interstitium. This finding was also observed in PC and an independent test PC cohort. Therefore, we propose that a combination of α-SMA and MVD can, in part, be a good indicator of MVI (CD34^+^/α-SMA^+^); that MVI is more critical than MVD alone in development of microvascular invasion; and that MVI is critical for further understanding possible underlying mechanisms of antiangiogenesis as an accelerator of tumor metastasis.

A recognized human EC marker is CD34. There have been reports in a mouse model that α-SMA and CD31 of an EC marker can coexpress in vascular walls [24], [26]; however, this is unclear in human. Our work confirms this α-SMA and CD34 coexpression pattern (see schematic diagram in the Fig. S7 in File S1). Those perivascular α-SMA-positive cells that wrap around ECs were identified as PVCs, and the PVC density was used as an indicator of MVI. Before this study, a big challenge was to select an appropriate molecular marker of PVCs. Expression of various markers has been reported in PVCs; i.e., NG2, platelet-derived growth factor receptor-beta (PDGFRβ), α-SMA, desmin, and RGS5 [23], and their expression might be tissue specific. Despite some PVC markers of HCC and PC having been identified in animal experiments [25], [26], [36], [37], study of their expression in human biopsies has largely been unexplored. Our results suggest that α-SMA is a suitable marker for MVI, because of its positive-staining rate and colocalized expression with CD34. Therefore, our findings establish that it is a marker of PVCs. This is consistent with Feig et al. [13] who considered that α-SMA-positive stromal fibroblasts might likely represent PVCs. For although NG2 could be stained positively in mouse specimens [38], investigation of it and other predictor molecules were failed in human tissues collected in our institute (see Fig. S8 in File S1).

Our results also demonstrate that in patients with high tumor MVD, as long as their MVI is also high, the metastatic probability could be low, and in this case, the prognosis would be fine. In contrast, the prognosis could be poor for patients with low MVD and also low MVI. Poor integrity of vessels provides numerous avenues for tumor cell intra/extravasation from the leaky vascular architecture, and those cells could transplant into target organs, resulting in metastasis. What makes matters worse is that in areas of vascular collapse, arising from poor PVC coverage, dramatically reduced tumor blood perfusion could result and produce regional hypoxia [19]. The resultant hypoxia-endowed tumor cells would have migratory and invasive properties through HIF-1α-induced epithelial-mesenchymal transition [22], [32], [39]. This situation would create a hostile tumor milieu where these cells could easily invade through the abnormal vessels and form tumor thrombi. This hypothesis is supported by an excellent study showing that depletion of pericytes causes tumor hypoxia and metastasis [22], and by our previous study showing that enhancement of MVI alleviates hypoxia and inhibits metastasis [38]. These findings are all consistent with our clinical observations. The present work also examines hypoxia biomarkers HIF-1α and CA IX intensities according to combinatorial marker groups of different MVI, and we found their indeed presents a gradient distribution. In both low MVD groups I and II, and in both high MVD groups III and IV, the higher MVI correlates with lower HIF-1α and CA IX. How MVI is regulated is unclear. Mazzone et al. reported that hypoxia and the HIF-1α/−2α-associated PHD2 protein could damage MVI [24], and it has been found that gene targeting of *Phd2* results in enhanced MVI and improved tumor response to chemotherapy [40]. The latter observation suggests that MVI could be regulated by hypoxia/HIF-1α via a feedback pathway. However, we do not have an independent prognostic value for PVCs marked by α-SMA, which is different from the findings of Cooke and colleagues [22]. Possible reasons for the discrepancy may be the heterogeneity of various tumors, or the potential lack of specificity of α-SMA. Our results imply that consideration of PVCs alone is insufficient; the key point, rather, lies in the association of PVCs with ECs.

Recent emerging data, both from our group and others, has revealed that vascular endothelial growth factor or vascular endothelial growth factor receptor (VEGFR) blockade by sorafenib or sunitinib leads to enhanced metastasis [20], [21], [41]. There are some insights into the mechanism of this phenomenon, but it is still not fully understood. We speculate that it is probably because antiangiogenesis targets both EC and PVC, as sunitinib can block VEGFR2 and PDGFRs [42]. The targeting of EC causes decreased MVD and diminished tumor growth, while targeting of PVC impairs MVI and elicits metastasis. Therefore, antiangiogenesis treatments that reduce MVD without protecting MVI could generate prometastatic effects. Collectively, our findings provide a monitoring tool for antiangiogenesis strategy. In addition, this tool might assist in evaluation of patients with superior tumor vasculature, as entrance criteria for cytotoxic adjuvant therapy.

Since Jain et al. [43] proposed the “tumor vascular normalization” hypothesis in 2001, accumulating evidence has gradually confirmed this viewpoint [19]. However, how to monitor vascular morphogenesis in clinical applications is still a big challenge. Batchelor et al. [44] applied magnetic resonance imaging for angiography; however, the results were inconsistent. Other techniques such as scanning electron microscopy or fluorescent dye perfusion [24] would be impractical for use in patients. From our findings, we suggest α-SMA and CD34, as dual marker for PVC, in combination with EC, as marker for vascular morphology, as perhaps the most convenient way to evaluate MVI.

In conclusion, using the two typical highly malignant tumor types HCC and PC, we observed that high tumor MVD, coupled with poor PVC coverage, is predictive of the worst prognosis. Using MVD as sole marker of angiogenesis is suboptimal as a prognostic factor. The conceptual framework of MVI is an important complement to MVD, and maintenance of MVI has the significant clinical benefit of preventing postoperative tumor recurrence and metastasis.

## Supporting Information

File S1 **Table S1–Table S11, Figure S1–S8. Table S1.** Clinicopathological features of 305 hepatocellular carcinoma patients from cohort 1. **Table S2.** Clinicopathological features of 57 pancreatic cancer patients from cohort 2. **Table S3.** Clinicopathological features of 52 pancreatic cancer patients from an independent test cohort 3^*^. **Table S4.** Relationship between tumor α-SMA-positive cell density and microvessel density and clinicopathological features of 305 hepatocellular carcinoma patients from cohort 1. **Table S5.** Relationship between tumor α-SMA-positive cell density and microvessel density and clinicopathological features of 57 pancreatic cancer patients from cohort 2. **Table S6.** Univariate and multivariate analyses of factors associated with survival and recurrence in 305 hepatocellular carcinoma patients from cohort 1. **Table S7.** Univariate and multivariate analyses of factors associated with survival and recurrence in 57 pancreatic cancer patients from cohort 2. **Table S8.** Univariate and multivariate analyses of factors associated with survival and recurrence in the small-tumor hepatocellular carcinoma subgroup^*^ from cohort 1. **Table S9.** Clinicopathological features of three cohorts of patients with hepatocellular carcinoma and pancreatic cancer of different subgroups of alpha-smooth muscle actin and microvessel density. **Table S10.** Prognostic values of variables for death and disease recurrence by receiver operating characteristic analysis of 305 hepatocellular carcinoma patients from cohort 1. **Table S11.** Prognostic values of variables for death and disease recurrence by receiver operating characteristic analysis of 57 pancreatic cancer patients from cohort 2. **Figure S1. The distributional characteristics of histograms for (A, C) α-SMA-positive cell density and (B, D) microvessel density (MVD) of each patient.** The cutoff point of α-SMA density and MVD-CD34 for definition of subgroups was the median value. SD, standard deviation. **Figure S2. Expression of alpha-smooth muscle actin (α-SMA) and CD34 in peritumoral normal (A, B) liver or (C, D) pancreas tissue (×200).** Compared with the heterogeneous intratumoral vessel distribution, the vascular morphology in peritumoral tissue was homogeneous. **Figure S3. Coexpression of (A) alpha-smooth muscle actin (α-SMA) and (B) CD34 in peritumoral normal pancreas tissue by immunohistochemical staining in serial sections (×200). Figure S4. Co-distribution of perivascular stromal cells and endothelial cells on tumor vascular wall by immunofluorescent double staining for alpha-smooth muscle actin (α-SMA) and CD34 in (A) hepatocellular carcinoma (HCC) and (B) pancreatic cancer (PC) (laser confocal microscopy, ×250). Figure S5. Cumulative recurrence-free survival curves of patients with low or high tumor (A) alpha-smooth muscle actin (α-SMA) density or (B) microvessel density (MVD) and (C) their combination in the 2-year recurrence subgroup of hepatocellular carcinoma (see Results for details).** Figures were not shown for the late recurrence subgroup. **Figure S6. Cumulative overall survival (OS) and recurrence-free survival (RFS) curves of patients with low or high tumor alpha-smooth muscle actin (α-SMA) density or microvessel density (MVD) and their combination in the small hepatocellular carcinoma (HCC) subgroup (maximum diameter of ≤5 cm;** ***n*** **= 179).** (A, B) The α-SMA density was associated with neither OS nor RFS. (C, D) Low MVD was associated with prolonged OS and RFS. (E, F) Patients were classified into four groups according to the combination of α-SMA density and MVD. Group II had the best OS and RFS, while group III had the worst OS and RFS. **Figure S7. Schematic diagram of distribution characteristics of alpha-smooth muscle actin (α-SMA)-positive stromal cells and CD34 in tumor tissue.** (Merged A and B) Immunofluorescent double staining of α-SMA and CD34 in frozen sections, showing an α-SMA^+^ (red) cell wrapping around a CD34^+^ (green) cell on the vascular wall (×750). **Figure S8. Schematic expression of NG2 and PDGFRβ in hepatocellular carcinoma (HCC) and pancreatic cancer (PC).** Rectangle shows the typical location of markers in perivascular cells (PVCs). Unfortunately, (A) NG2 staining reveals non-PVC-specific expression in HCC; and (C) almost negative expression in PC (as indicated by arrow). These results were obtained with four different antibodies (Millipore, R&D, Abcam, and Santa Cruz), all employing the same immunohistochemistry protocol. (B, D) PDGFRβ staining also showed positive expression in tumor cell nuclei (as indicated by arrow); hence, it could not be used as a marker in quantitative analysis of PVCs. Moreover, neither NG2 nor PDGFRβ was found to be associated with patient outcome, either separately or in cooperation with MVD.
(DOC)
