# Supplementary material for: Intratumoral α-SMA Enhances the Prognostic Potency of CD34 Associated with Maintenance of Microvessel Integrity in Hepatocellular Carcinoma and Pancreatic Cancer
Source: PLoS One. 2013 Aug 5;8(8):e71189. doi: 10.1371/journal.pone.0071189 (PMC3734294; doi:10.1371/journal.pone.0071189)
Supplement: File S1 — Table S1–Table S11, Figure S1–S8. Table S1. Clinicopathological features of 305 hepatocellular carcinoma patients from cohort 1. Table S2. Clinicopathological features of 57 pancreatic cancer patients from cohort 2. Table S3. Clinicopathological features of 52 pancreatic cancer patients from an independent test cohort 3*. Table S4. Relationship between tumor α-SMA-positive cell density and microvessel density and clinicopathological features of 305 hepatocellular carcinoma patients from cohort 1. Table S5. Relationship between tumor α-SMA-positive cell density and microvessel density and clinicopathological features of 57 pancreatic cancer patients from cohort 2. Table S6. Univariate and multivariate analyses of factors associated with survival and recurrence in 305 hepatocellular carcinoma patients from cohort 1. Table S7. Univariate and multivariate analyses of factors associated with survival and recurrence in 57 pancreatic cancer patients from cohort 2. Table S8. Univariate and multivariate analyses of factors associated with survival and recurrence in the small-tumor hepatocellular carcinoma subgroup* from cohort 1. Table S9. Clinicopathological features of three cohorts of patients with hepatocellular carcinoma and pancreatic cancer of different subgroups of alpha-smooth muscle actin and microvessel density. Table S10. Prognostic values of variables for death and disease recurrence by receiver operating characteristic analysis of 305 hepatocellular carcinoma patients from cohort 1. Table S11. Prognostic values of variables for death and disease recurrence by receiver operating characteristic analysis of 57 pancreatic cancer patients from cohort 2. Figure S1. The distributional characteristics of histograms for (A, C) α-SMA-positive cell density and (B, D) microvessel density (MVD) of each patient. The cutoff point of α-SMA density and MVD-CD34 for definition of subgroups was the median value. SD, standard deviation. Figure S2. Expression of alpha-smooth muscle [file pone.0071189.s001.doc]

**Supporting Information**

**Table S1.** Clinicopathological features of 305 hepatocellular carcinoma patients from cohort 1.

| Features | Values/Counts (*n* = 305) |
| --- | --- |
| Age (years, median (range)) | 52 (22–80) |
| Gender (male/female) | 255/50 |
| Preoperative ALT (U/L, median (range)) | 44 (0–806) |
| α-Fetoprotein (ng/ml, median (range)) | 176 (0–60500) |
| Hepatitis (years, median (range)) | 10 (0–48) |
| Liver cirrhosis (yes/no) | 72/233 |
| Hepatitis B history (yes/no) | 261/44 |
| Hepatitis B e antigen (positive/negative) | 117/188 |
| Tumor size (cm, mean ± SD) | 5.60 ± 3.90 |
| Encapsulation (complete/no) | 149/156 |
| Tumor differentiation (Stage I–II/Stage III–IV) | 214/91 |
| Intrahepatic metastasis (yes/no) | 45/260 |
| Microvascular invasion (yes/no) | 126/179 |
| UICC TNM stage (I/II/IIIA) | 36/129/140 |

Abbreviations: ALT, alanine aminotransferase; SD, standard deviation; UICC, International Union against Cancer Classification.

**Table S2.** Clinicopathological features of 57 pancreatic cancer patients from cohort 2.

| Features | Values/Counts (*n* = 57) |
| --- | --- |
| Age (years, median (range)) | 61 (39–77) |
| Gender (male/female) | 32/25 |
| Preoperative CA199 (U/mL, median (range)) | 153.70 (0–2085) |
| Preoperative CA50 (U/mL, median (range)) | 67.24 (0–1100.78) |
| Preoperative CA242 (U/mL, median (range)) | 35.20 (0–234.40) |
| Hepatitis B history (yes/no) | 10/47 |
| Tumor size (cm, mean ± SD) | 3.86 ± 1.78 |
| Tumor differentiation (Grade 1–2/Grade 3–4) | 24/33 |
| Nodal involvement (yes/no) | 10/47 |
| Microvascular invasion (yes/no) | 12/45 |
| Perineural invasion (yes/no) | 43/14 |
| UICC TNM stage (IB/IIA/IIB) | 11/22/24 |

Abbreviations: CA, carcinoembryonic antigen; SD, standard deviation; UICC, International Union against Cancer Classification.

**Table S3.** Clinicopathological features of 52 pancreatic cancer patients from an independent test cohort 3^*^.

| Features | Values/Counts (*n* = 52) |
| --- | --- |
| Age (years, median (range)) | 61 (39–77) |
| Gender (male/female) | 30/22 |
| Preoperative CA199 (U/mL, median (range)) | 108.10 (4.20–2084.00) |
| Tumor size (cm, mean ± SD) | 3.97 ± 1.99 |
| Tumor differentiation (Grade 1–2/Grade 3–4) | 14/38 |
| Nodal involvement (yes/no) | 28/24 |
| Microvascular invasion (yes/no) | 13/39 |
| Perineural invasion (yes/no) | 41/11 |
| UICC TNM stage (IB/IIA/IIB) | 10/14/28 |

Abbreviations: CA, carcinoembryonic antigen; SD, standard deviation; UICC, International Union against Cancer Classification.

^*^Another independent test cohort including 52 cases with pathology-proven pancreatic cancer who underwent curative resection was collected. All patients were observed until June 2012, with a median follow-up time of 14.5 months. In the last follow-up, 33 patients had tumor recurrence and 20 died. The 0.5-, 1-, and 2-year overall survival rates were 88.5%, 67.3%, and 61.5%, respectively; and the 0.5-, 1-, and 2-year recurrence rates were 25%, 55.8%, and 63.5%, respectively.

**Table S4.** Relationship between tumor α-SMA-positive cell density and microvessel density and clinicopathological features of 305 hepatocellular carcinoma patients from cohort 1.

| Variables | α-SMA Density | | | | | MVD | | | | |
| --- | --- | --- | --- | --- | --- | --- | --- | --- | --- | --- |
|  | High (n = 153) | | Low (n = 152) | | *P* | High (n = 153) | | Low (n = 152) | | *P* |
|  | No. of Patients | % | No. of Patients | % |  | No. of Patients | % | No. of Patients | % |  |
| Age, years^*^ | 50.88±10.34 | | 53.41±11.59 | | .044 | 53.12±12.22 | | 51.15±9.64 | | .118^†^ |
| Gender |  |  |  |  | .116 |  |  |  |  | .340 |
| Male | 133 | 87 | 122 | 80 |  | 131 | 86 | 124 | 82 |  |
| Female | 20 | 13 | 30 | 20 |  | 22 | 14 | 28 | 18 |  |
| Hepatitis, years^*^ | 11.44±14.15 | | 12.63±15.81 | | .493 | 11.68±14.84 | | 12.39±15.18 | | .681 |
| Hepatitis B history |  |  |  |  | .530 |  |  |  |  | .108 |
| Yes | 129 | 84 | 132 | 87 |  | 126 | 82 | 135 | 89 |  |
| No | 24 | 16 | 20 | 13 |  | 27 | 18 | 17 | 11 |  |
| HBeAg |  |  |  |  | .385 |  |  |  |  | .085 |
| Positive | 55 | 36 | 62 | 41 |  | 66 | 43 | 51 | 34 |  |
| Negative | 98 | 64 | 90 | 59 |  | 87 | 57 | 101 | 66 |  |
| ALT, U/L^*^ | 58.99±74.41 | | 56.03±36.46 | | .659 | 61.80±74.89 | | 53.25±35.42 | | .203 |
| AFP, ng/dl^*^ | 5578.41±  14991.00 | | 5588.57±  13849.30 | | .995 | 7667.41±  17286.92 | | 3485.82±  10410.35 | | .011^†^ |
| Liver cirrhosis |  |  |  |  | .029 |  |  |  |  | .763 |
| Yes | 28 | 18 | 44 | 29 |  | 35 | 23 | 37 | 24 |  |
| No | 125 | 82 | 108 | 71 |  | 118 | 77 | 115 | 76 |  |
| Tumor size, cm^*^ | 5.28±3.37 | | 5.92±4.36 | | .151^†^ | 6.79±4.66 | | 4.40±2.44 | | .000^†^ |
| Intrahepatic metastasis |  |  |  |  | .001 |  |  |  |  | .007 |
| Yes | 12 | 8 | 33 | 22 |  | 31 | 20 | 14 | 9 |  |
| No | 141 | 92 | 119 | 78 |  | 122 | 80 | 138 | 91 |  |
| Tumor encapsulation |  |  |  |  | .391 |  |  |  |  | .865 |
| Complete | 71 | 46 | 78 | 51 |  | 74 | 48 | 75 | 49 |  |
| No | 82 | 54 | 74 | 49 |  | 79 | 52 | 77 | 51 |  |
| Microvascular invasion |  |  |  |  | .018 |  |  |  |  | .003 |
| Yes | 53 | 35 | 73 | 48 |  | 76 | 50 | 50 | 33 |  |
| No | 100 | 65 | 79 | 52 |  | 77 | 50 | 102 | 67 |  |
| Tumor differentiation |  |  |  |  | .008 |  |  |  |  | .066 |
| Stage I–II | 118 | 77 | 96 | 63 |  | 100 | 65 | 114 | 75 |  |
| Stage III–IV | 35 | 23 | 56 | 37 |  | 53 | 35 | 38 | 25 |  |
| TNM stage |  |  |  |  | .074 |  |  |  |  | .044 |
| I | 21 | 14 | 15 | 10 |  | 13 | 9 | 23 | 15 |  |
| II | 55 | 36 | 74 | 49 |  | 60 | 39 | 69 | 45 |  |
| IIIA | 77 | 50 | 63 | 41 |  | 80 | 52 | 60 | 40 |  |

^*^Mean ± standard deviation, Student’s *t*-test.

^†^Equal variances not assumed.

*p* < 0.05 was considered statistically significant.

Abbreviations: α-SMA, alpha-smooth muscle actin; MVD, microvessel density; HBeAg, hepatitis B e antigen; ALT, alanine aminotransferase; AFP, α-fetoprotein; TNM, tumor-node-metastasis.

**Table S5.** Relationship between tumor α-SMA-positive cell density and microvessel density and clinicopathological features of 57 pancreatic cancer patients from cohort 2.

| Variables | α-SMA Density | | | | | MVD | | | | |
| --- | --- | --- | --- | --- | --- | --- | --- | --- | --- | --- |
|  | High (n = 29) | | Low (n = 28) | | *P* | High (n = 28) | | Low (n = 29) | | *P* |
|  | No. of Patients | % | No. of Patients | % |  | No. of Patients | % | No. of Patients | % |  |
| Age, years^*^ | 59.62±8.96 | | 62.14±7.87 | | .264 | 60.29±8.30 | | 61.41±8.73 | | .619 |
| Gender |  |  |  |  | .080 |  |  |  |  | .494 |
| Male | 13 | 45 | 19 | 68 |  | 17 | 61 | 15 | 52 |  |
| Female | 16 | 55 | 9 | 32 |  | 11 | 39 | 14 | 48 |  |
| Tumor size, cm^*^ | 3.75±1.51 | | 3.98±2.04 | | .629 | 3.49±1.51 | | 4.23±1.96 | | .118 |
| Tumor size, group |  |  |  |  | .896 |  |  |  |  | .024 |
| ≤3 cm (n = 28) | 14 | 48 | 14 | 50 |  | 18 | 64 | 10 | 35 |  |
| >3 cm (n = 29) | 15 | 52 | 14 | 50 |  | 10 | 36 | 19 | 65 |  |
| Nodal involvement |  |  |  |  | .046 |  |  |  |  | .011 |
| Yes | 8 | 28 | 15 | 54 |  | 16 | 57 | 7 | 24 |  |
| No | 21 | 72 | 13 | 46 |  | 12 | 43 | 22 | 76 |  |
| Perineural invasion |  |  |  |  | .589 |  |  |  |  | .940 |
| Yes | 21 | 72 | 22 | 79 |  | 21 | 75 | 22 | 76 |  |
| No | 8 | 28 | 6 | 21 |  | 7 | 25 | 7 | 24 |  |
| Microvascular invasion |  |  |  |  | .044^†^ |  |  |  |  | .044^†^ |
| Yes | 3 | 10 | 9 | 32 |  | 9 | 32 | 3 | 10 |  |
| No | 26 | 90 | 19 | 68 |  | 19 | 68 | 26 | 90 |  |
| CA199, U/mL^*^ | 458.55±579.93 | | 622.15±806.70 | | .385^‡^ | 591.20±719.58 | | 488.43±687.59 | | .584 |
| CA50, U/mL^*^ | 155.16±223.45 | | 224.47±285.78 | | .311 | 209.11±270.37 | | 169.99±244.67 | | .569 |
| CA242, U/mL^*^ | 61.81±62.54 | | 80.46±87.64 | | .361^‡^ | 76.78±75.81 | | 65.37±76.72 | | .575 |
| Hepatitis B history |  |  |  |  | .179^†^ |  |  |  |  | .504^†^ |
| Yes | 3 | 10 | 7 | 25 |  | 6 | 21 | 4 | 14 |  |
| No | 26 | 90 | 21 | 75 |  | 22 | 79 | 25 | 86 |  |
| Tumor differentiation |  |  |  |  | .042 |  |  |  |  | .236 |
| Grade 1–2 | 16 | 55 | 8 | 29 |  | 14 | 50 | 10 | 34 |  |
| Grade 3–4 | 13 | 45 | 20 | 71 |  | 14 | 50 | 19 | 66 |  |
| TNM stage |  |  |  |  | .024 |  |  |  |  | .085 |
| IB and IIA | 21 | 72 | 12 | 43 |  | 13 | 46 | 20 | 69 |  |
| IIB | 8 | 28 | 16 | 57 |  | 15 | 54 | 9 | 31 |  |

^*^Mean ± standard deviation, Student’s *t*-test.

^†^Twenty-five percent of all the cells have expected count less than 5; Fisher’s exact test.

^‡^Equal variances not assumed.

*p* < 0.05 was considered statistically significant.

Abbreviations: α-SMA, alpha-smooth muscle actin; MVD, microvessel density; CA, carcinoembryonic antigen; TNM, tumor-node-metastasis.

**Table S6.** Univariate and multivariate analyses of factors associated with survival and recurrence in 305 hepatocellular carcinoma patients from cohort 1.

| Factors | OS | | | | RFS | | | |
| --- | --- | --- | --- | --- | --- | --- | --- | --- |
|  | Univariate *P* | Multivariate | | | Univariate *P* | Multivariate | | |
|  |  | HR | 95% CI | *P* |  | HR | 95% CI | *P* |
| Age: < 51 vs. ≥ 51 years | .173 |  |  | NA | .536 |  |  | NA |
| Gender: female vs. male | .337 |  |  | NA | .405 |  |  | NA |
| Hepatitis B history: no vs. yes | .149 |  |  | NA | .671 |  |  | NA |
| HBeAg: negative vs. positive | .177 |  |  | NA | .006 | 1.443 | 1.019–2.042 | .039 |
| Liver cirrhosis: no vs. yes | .146 |  |  | NA | .180 |  |  | NA |
| ALT: ≤ 75 vs. > 75 U/L | .692 |  |  | NA | .433 |  |  | NA |
| AFP: ≤ 300 vs. > 300 ng/dl | .087 |  |  | NA | .074 |  |  | NA |
| Tumor size: ≤ 5 vs. > 5 cm | < .001 | 2.435 | 1.588–3.736 | < .001 | < .001 | 1.594 | 1.097–2.315 | .014 |
| Tumor differentiation: Stages I–II vs. III–IV | .001 | 1.661 | 1.112–2.482 | .013 | .011 |  |  | NS |
| Tumor number: single vs. multiple | .006 |  |  | NS | .001 |  |  | NS |
| Tumor encapsulation: no vs. complete | .103 |  |  | NA | .411 |  |  | NA |
| Microvascular invasion: no vs. yes | < .001 |  |  | NS | .001 |  |  | NS |
| Intrahepatic metastasis: no vs. yes | < .001 | 1.875 | 1.193–2.947 | .006 | < .001 | 2.055 | 1.320–3.200 | .001 |
| TNM stage: I vs. II vs. IIIA | < .001 | 0.368 | 0.128–1.063 | .018 | < .001 | 0.512 | 0.265–0.989 | < .001 |
| Intratumoral α-SMA density: low vs. high | .071 |  |  | NS | .079 |  |  | NS |
| Intratumoral MVD: low vs. high | < .001 | 4.236 | 2.659–6.747 | < .001 | < .001 | 2.585 | 1.784–3.745 | < .001 |
| Combine α-SMA density and MVD^*,†^ | < .001 | 1.381 | 0.830–2.296 | < .001 | < .001 | 1.325 | 0.801–2.191 | < .001 |

^*^ Patients were classified into four groups according to their intratumoral α-SMA density and MVD: group I (*n* = 68), both low density; group II (*n* = 84), high α-SMA density and low MVD; group III (*n* = 86), low α-SMA density but high MVD; and group IV (*n* = 67), both high density.

^†^ The multivariate analysis of different subgroups of α-SMA and MVD was analyzed together with other risk factors identified by univariate analysis but excluding α-SMA and MVD themselves, in order to avoiding the interference of them on the combinational group.

Abbreviations: OS, overall survival; RFS, recurrence free survival; HR, Hazard Ratio; CI, confidence interval; HBeAg, hepatitis B e antigen; ALT, alanine aminotransferase; AFP, α-fetoprotein; TNM, tumor-node-metastasis; α-SMA, alpha-smooth muscle actin; MVD, microvessel density; NA, not adapted; NS, not significant.

**Table S7.** Univariate and multivariate analyses of factors associated with survival and recurrence in 57 pancreatic cancer patients from cohort 2.

| Factors | OS | | | | RFS | | | |
| --- | --- | --- | --- | --- | --- | --- | --- | --- |
|  | Univariate *P* | Multivariate | | | Univariate *P* | Multivariate | | |
|  |  | HR | 95% CI | *P* |  | HR | 95% CI | *P* |
| Age: < 61 vs. ≥ 61 years | .579 |  |  | NA | .887 |  |  | NA |
| Gender: female vs. male | .255 |  |  | NA | .334 |  |  | NA |
| Tumor size: ≤ 3 vs. > 3 cm | .751 |  |  | NA | .431 |  |  | NA |
| Nodal involvement: no vs. yes | .724 |  |  | NA | .077 |  |  | NS |
| Perineural invasion: no vs. yes | .465 |  |  | NA | .297 |  |  | NA |
| Microvascular invasion: no vs. yes | .018 |  |  | NS | .001 | 3.267 | 1.375–7.764 | .007 |
| CA199: ≤ 75 vs. > 75 U/mL | .918 |  |  | NA | .304 |  |  | NA |
| CA50: ≤ 75 vs. > 75 U/mL | .046 |  |  | NS | .399 |  |  | NA |
| CA242: ≤ 75 vs. > 75 U/mL | .034 |  |  | NS | .698 |  |  | NA |
| Platelet: ≤ 200 vs. > 200 × 10^9^/L | .730 |  |  | NA | .108 |  |  | NA |
| Hepatitis B history: no vs. yes | .031 |  |  | NS | .611 |  |  | NA |
| Tumor differentiation: Grades 1-2 vs. 3-4 | .019 | 0.139 | 0.039–0.494 | .002 | .918 |  |  | NA |
| TNM stage: IB and IIA vs. IIB | .250 |  |  | NA | .389 |  |  | NA |
| Intratumoral α-SMA density: low vs. high | .072 |  |  | NS | .107 |  |  | NS |
| Intratumoral MVD: low vs. high | .046 | 3.578 | 1.247–10.265 | .018 | .008 | 2.230 | 1.061–4.688 | .034 |
| Combine α-SMA density and MVD^*,†^ | .026 | 6.294 | 1.224–32.357 | .028 | < .001 | 2.534 | 0.866–7.411 | .009 |

^*^ Patients were classified into four groups according to their intratumoral α-SMA density and MVD: group I (*n* = 14), both low density; group II (*n* = 15), high α-SMA density and low MVD; group III (*n* = 13), low α-SMA density but high MVD; and group IV (*n* = 15), both high density.

^†^ The multivariate analysis of different subgroups of α-SMA and MVD was analyzed together with other risk factors identified by univariate analysis but excluding α-SMA and MVD themselves, in order to avoiding the interference of them on the combinational group.

Abbreviations: OS, overall survival; RFS, recurrence free survival; HR, Hazard Ratio; CI, confidence interval; TNM, tumor-node-metastasis; α-SMA, alpha-smooth muscle actin; MVD, microvessel density; NA, not adapted; NS, not significant.

**Table S8.** Univariate and multivariate analyses of factors associated with survival and recurrence in the small-tumor hepatocellular carcinoma subgroup^*^ from cohort 1.

| Factors | OS | | | | RFS | | | |
| --- | --- | --- | --- | --- | --- | --- | --- | --- |
|  | Univariate *P* | Multivariate | | | Univariate *P* | Multivariate | | |
|  |  | HR | 95% CI | *P* |  | HR | 95% CI | *P* |
| Age: < 51 vs. ≥ 51 years | .443 |  |  | NA | .172 |  |  | NA |
| Gender: female vs. male | .338 |  |  | NA | .840 |  |  | NA |
| Hepatitis B history: no vs. yes | .269 |  |  | NA | .520 |  |  | NA |
| HBeAg: negative vs. positive | .098 |  |  | NA | .001 | 2.152 | 1.297–3.570 | .003 |
| Liver cirrhosis: no vs. yes | .830 |  |  | NA | .866 |  |  | NA |
| ALT: ≤ 75 vs. > 75 U/L | .563 |  |  | NA | .978 |  |  | NA |
| AFP: ≤ 300 vs. > 300 ng/dl | .663 |  |  | NA | .602 |  |  | NA |
| Tumor size: ≤ 3 vs. > 3 cm | .058 |  |  | NA | .076 |  |  | NA |
| Tumor differentiation: Stages I–II vs. III–IV | .161 |  |  | NA | .021 |  |  | NS |
| Tumor number: single vs. multiple | .087 |  |  | NA | .048 |  |  | NS |
| Tumor encapsulation: no vs. complete | .804 |  |  | NA | .556 |  |  | NA |
| Microvascular invasion: no vs. yes | .001 | 2.095 | 1.083–4.051 | .028 | .031 |  |  | NS |
| Intrahepatic metastasis: no vs. yes | .003 | 2.511 | 1.135–5.557 | .023 | < .001 | 2.964 | 1.567–5.606 | .001 |
| TNM stage: I vs. II vs. IIIA | .009 |  |  | NS | .009 | 0.494 | 0.248–0.986 | .023 |
| Intratumoral α-SMA density: low vs. high | .520 |  |  | NS | .153 |  |  | NS |
| Intratumoral MVD: low vs. high | < .001 | 5.776 | 2.583–12.919 | < .001 | .001 | 2.100 | 1.260–3.500 | .004 |
| Combine α-SMA density and MVD^†,‡^ | < .001 | 1.167 | 0.532–2.562 | < .001 | < .001 | 1.495 | 0.692–3.229 | .009 |

^*^ Maximum diameter ≤ 5 cm, *n* = 179.

^†^ Patients were classified into four groups according to their intratumoral α-SMA density and MVD: group I (*n* = 44), both low density; group II (*n* = 60), high α-SMA density and low MVD; group III (*n* = 41), low α-SMA density but high MVD; and group IV (*n* = 34), both high density.

^‡^ The multivariate analysis of different subgroups of α-SMA and MVD was analyzed together with other risk factors identified by univariate analysis but excluding α-SMA and MVD themselves, in order to avoiding the interference of them on the combinational group.

Abbreviations: OS, overall survival; RFS, recurrence free survival; HR, Hazard Ratio; CI, confidence interval; HBeAg, hepatitis B e antigen; ALT, alanine aminotransferase; AFP, α-fetoprotein; TNM, tumor-node-metastasis; α-SMA, alpha-smooth muscle actin; MVD, microvessel density; NA, not adapted; NS, not significant.

**Table S9.** Clinicopathological features of three cohorts of patients with hepatocellular carcinoma and pancreatic cancer of different subgroups of alpha-smooth muscle actin and microvessel density.

| Features | Values/Counts | | | |
| --- | --- | --- | --- | --- |
|  | group I | group II | group III | group IV |
| HCC (Cohort 1^*^, *n* = 305) |  |  |  |  |
| Age (years, median (range)) | 53 (31–78) | 49 (26–71) | 53 (22–80) | 54 (28–77) |
| Gender (male/female) | 54/14 | 70/14 | 72/14 | 59/8 |
| Preoperative ALT (U/L, median (range)) | 42 (9–126) | 45 (10–806) | 47 (0–202) | 55 (12–143) |
| α-Fetoprotein (ng/ml, median (range)) | 147 (0–60500) | 115 (0–60500) | 300 (0–60500) | 621 (1–60500) |
| Hepatitis (years, median (range)) | 10 (0–99) | 10 (0–99) | 10 (0–99) | 14 (0–99) |
| Liver cirrhosis (yes/no) | 22/46 | 15/69 | 20/66 | 15/52 |
| Hepatitis B history (yes/no) | 62/6 | 73/11 | 73/13 | 53/14 |
| Hepatitis B e antigen (positive/negative) | 43/25 | 58/26 | 48/38 | 39/28 |
| Tumor size (cm, mean ± SD) | 4.71 ± 2.75 | 4.15 ± 2.14 | 7.23 ± 5.04 | 6.23 ± 4.08 |
| Encapsulation (complete/no) | 38/30 | 37/47 | 40/46 | 34/33 |
| Tumor differentiation (Stage I–II/Stage III–IV) | 44/24 | 70/14 | 52/34 | 48/19 |
| Intrahepatic metastasis (yes/no) | 6/62 | 8/76 | 29/57 | 2/65 |
| Microvascular invasion (yes/no) | 18/50 | 32/52 | 57/29 | 19/48 |
| UICC TNM stage (I/II/IIIA) | 9/36/23 | 14/33/37 | 5/34/47 | 8/26/33 |
|  |  |  |  |  |
| PC (Cohort 2^*^, *n* = 57) |  |  |  |  |
| Age (years, median (range)) | 59 (44–76) | 62 (42–73) | 62 (54–77) | 61 (39–71) |
| Gender (male/female) | 6/8 | 9/6 | 10/3 | 7/8 |
| Preoperative CA199 (U/mL, median (range)) | 225.70 (0–1993) | 103.70 (0–2085) | 273 (0.80–2060) | 193.80 (0.80–2060) |
| Preoperative CA50 (U/mL, median (range)) | 64.29 (0–439.72) | 67.24 (0–1100.78) | 79.1 (7.17–871.63) | 75.18 (0–1039.17) |
| Preoperative CA242 (U/mL, median (range)) | 35.20 (0–150.60) | 28.10 (0–234.40) | 83.40 (0–199.90) | 39.60 (0–183.10) |
| Hepatitis B history (yes/no) | 1/13 | 3/12 | 4/9 | 2/13 |
| Tumor size (cm, mean ± SD) | 3.75 ± 1.07 | 4.67 ± 2.48 | 3.18 ± 0.95 | 3.75 ± 1.87 |
| Tumor differentiation (Grade 1–2/Grade 3–4) | 9/5 | 1/14 | 7/6 | 7/8 |
| Nodal involvement (yes/no) | 1/13 | 2/13 | 5/8 | 2/13 |
| Microvascular invasion (yes/no) | 1/13 | 2/13 | 7/6 | 2/13 |
| Perineural invasion (yes/no) | 10/4 | 12/3 | 10/3 | 11/4 |
| UICC TNM stage (IB/IIA/IIB) | 5/6/3 | 2/7/6 | 1/2/10 | 3/7/5 |
|  |  |  |  |  |
| PC (Cohort 3^*^, *n* = 52) |  |  |  |  |
| Age (years, median (range)) | 60 (42–71) | 59 (49–72) | 62 (42–73) | 60 (39–77) |
| Gender (male/female) | 7/7 | 5/7 | 10/2 | 8/6 |
| Preoperative CA199 (U/mL, median (range)) | 103.70 (9.10–2084) | 93.20 (5.90–2060) | 171.60 (4.20–2084) | 101.70 (8.20–2060) |
| Tumor size (cm, mean ± SD) | 3.79 ± 2.13 | 4.11 ± 2.43 | 4.33 ± 2.34 | 3.71 ± 1.04 |
| Tumor differentiation (Grade 1–2/Grade 3–4) | 12/2 | 9/3 | 9/3 | 8/6 |
| Nodal involvement (yes/no) | 7/7 | 5/7 | 10/2 | 6/8 |
| Microvascular invasion (yes/no) | 2/12 | 2/10 | 5/7 | 4/10 |
| Perineural invasion (yes/no) | 12/2 | 7/5 | 9/3 | 13/1 |
| UICC TNM stage (IB/IIA/IIB) | 2/5/7 | 4/3/5 | 0/2/10 | 4/4/6 |

^*^ Patients were classified into four groups according to their intratumoral α-SMA density and MVD: group I, both low density; group II, high α-SMA density and low MVD; group III, low α-SMA density but high MVD; and group IV, both high density.

Abbreviations: HCC, hepatocellular carcinoma; ALT, alanine aminotransferase; SD, standard deviation; UICC, International Union against Cancer Classification; PC, pancreatic cancer; CA, carcinoembryonic antigen.

**Table S10.** Prognostic values of variables for death and disease recurrence by receiver operating characteristic analysis of 305 hepatocellular carcinoma patients from cohort 1.

| Variables | Area under curve | 95% CI | *P* value |
| --- | --- | --- | --- |
| Death |  |  |  |
| Intratumoral α-SMA density | 0.544 | 0.477–0.612 | .201 |
| Intratumoral MVD | 0.707 | 0.646–0.767 | .000 |
| Combine α-SMA and MVD | 0.743 | 0.686–0.800 | .000 |
| Tumor size | 0.682 | 0.618–0.746 | .000 |
| Tumor differentiation | 0.577 | 0.509–0.645 | .026 |
| Intrahepatic metastasis | 0.601 | 0.532–0.670 | .004 |
| TNM stage | 0.695 | 0.634–0.755 | .000 |
| 2-year recurrence |  |  |  |
| Intratumoral α-SMA density | 0.528 | 0.463–0.594 | .399 |
| Intratumoral MVD | 0.592 | 0.528–0.656 | .006 |
| Combine α-SMA and MVD | 0.615 | 0.551–0.678 | .001 |
| HBeAg | 0.576 | 0.511–0.641 | .023 |
| Tumor size | 0.583 | 0.518–0.648 | .013 |
| Intrahepatic metastasis | 0.564 | 0.498–0.629 | .057 |
| TNM stage | 0.621 | 0.558–0.685 | .000 |

Abbreviations: ROC, receiver operating characteristic; CI, confidence interval; α-SMA, alpha-smooth muscle actin; MVD, microvessel density; TNM, tumor-node-metastasis; HBeAg, hepatitis B e antigen.

**Table S11.** Prognostic values of variables for death and disease recurrence by receiver operating characteristic analysis of 57 pancreatic cancer patients from cohort 2.

| Variables | Area under curve | 95% CI | *P* value |
| --- | --- | --- | --- |
| Death |  |  |  |
| Intratumoral α-SMA density | 0.641 | 0.493–0.789 | .075 |
| Intratumoral MVD | 0.692 | 0.550–0.835 | .015 |
| Combine α-SMA and MVD | 0.758 | 0.637–0.880 | .001 |
| Tumor differentiation | 0.823 | 0.705–0.942 | .000 |
| Microvascular invasion | 0.514 | 0.358–0.669 | .863 |
| 2-year recurrence |  |  |  |
| Intratumoral α-SMA density | 0.616 | 0.467–0.764 | .139 |
| Intratumoral MVD | 0.708 | 0.570–0.847 | .008 |
| Combine α-SMA and MVD | 0.790 | 0.666–0.913 | .000 |
| Nodal involvement | 0.525 | 0.372–0.677 | .753 |
| Microvascular invasion | 0.574 | 0.425–0.723 | .344 |

Abbreviations: ROC, receiver operating characteristic; CI, confidence interval; α-SMA, alpha-smooth muscle actin; MVD, microvessel density.


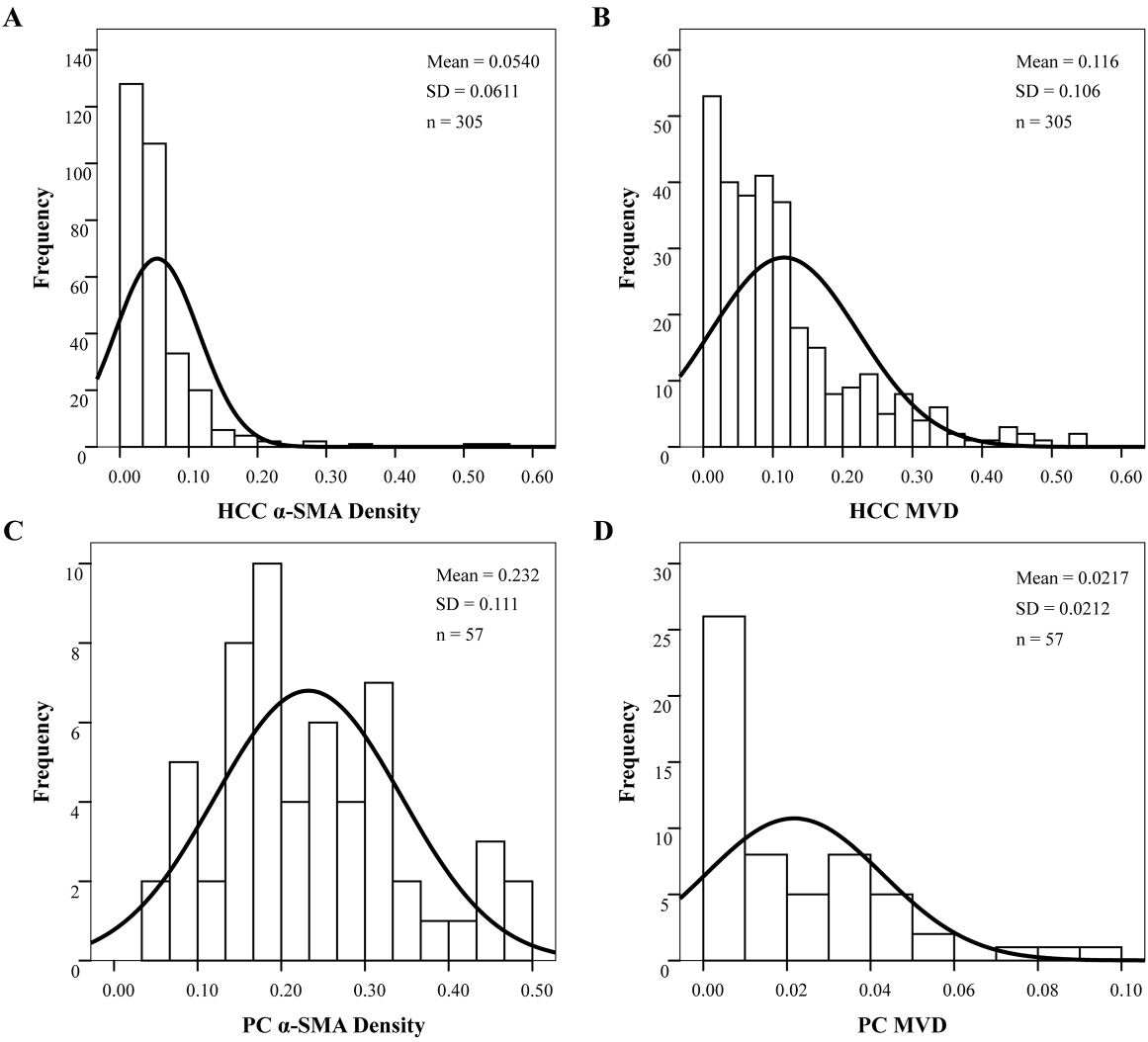


**Figure S1.** The distributional characteristics of histograms for (A, C) α-SMA-positive cell density and (B, D) microvessel density (MVD) of each patient. The cutoff point of α-SMA density and MVD-CD34 for definition of subgroups was the median value. SD, standard deviation.


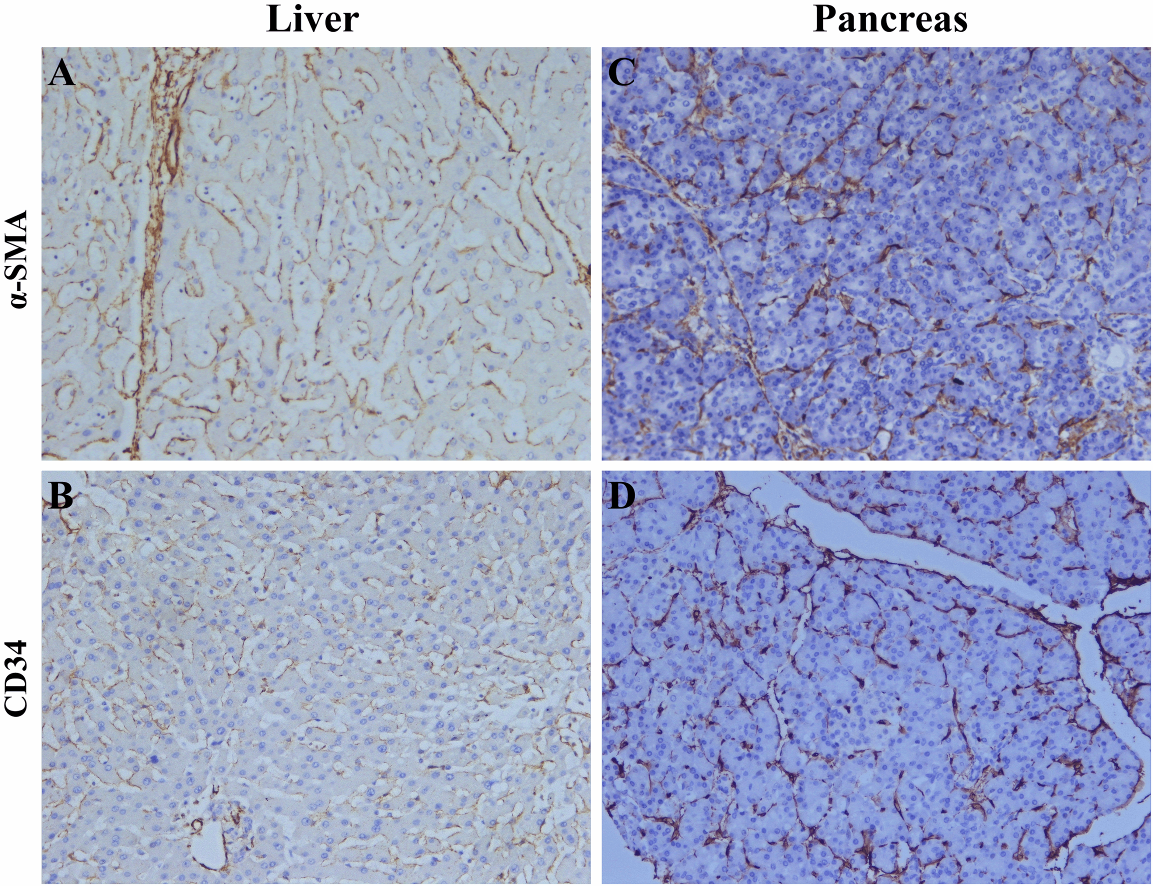


**Figure S2.** Expression of alpha-smooth muscle actin (α-SMA) and CD34 in peritumoral normal (A, B) liver or (C, D) pancreas tissue (×200). Compared with the heterogeneous intratumoral vessel distribution, the vascular morphology in peritumoral tissue was homogeneous.


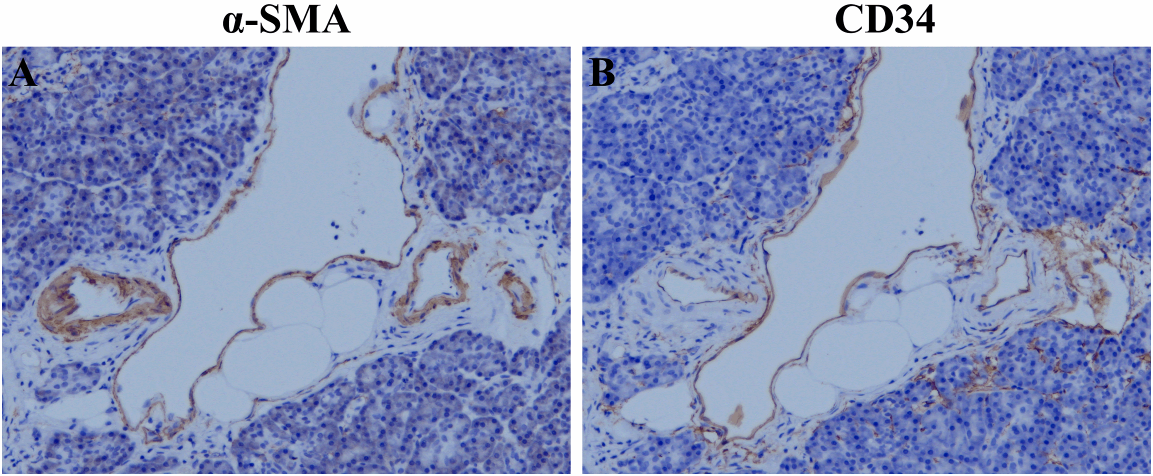


**Figure S3.** Coexpression of (A) alpha-smooth muscle actin (α-SMA) and (B) CD34 in peritumoral normal pancreas tissue by immunohistochemical staining in serial sections (×200).


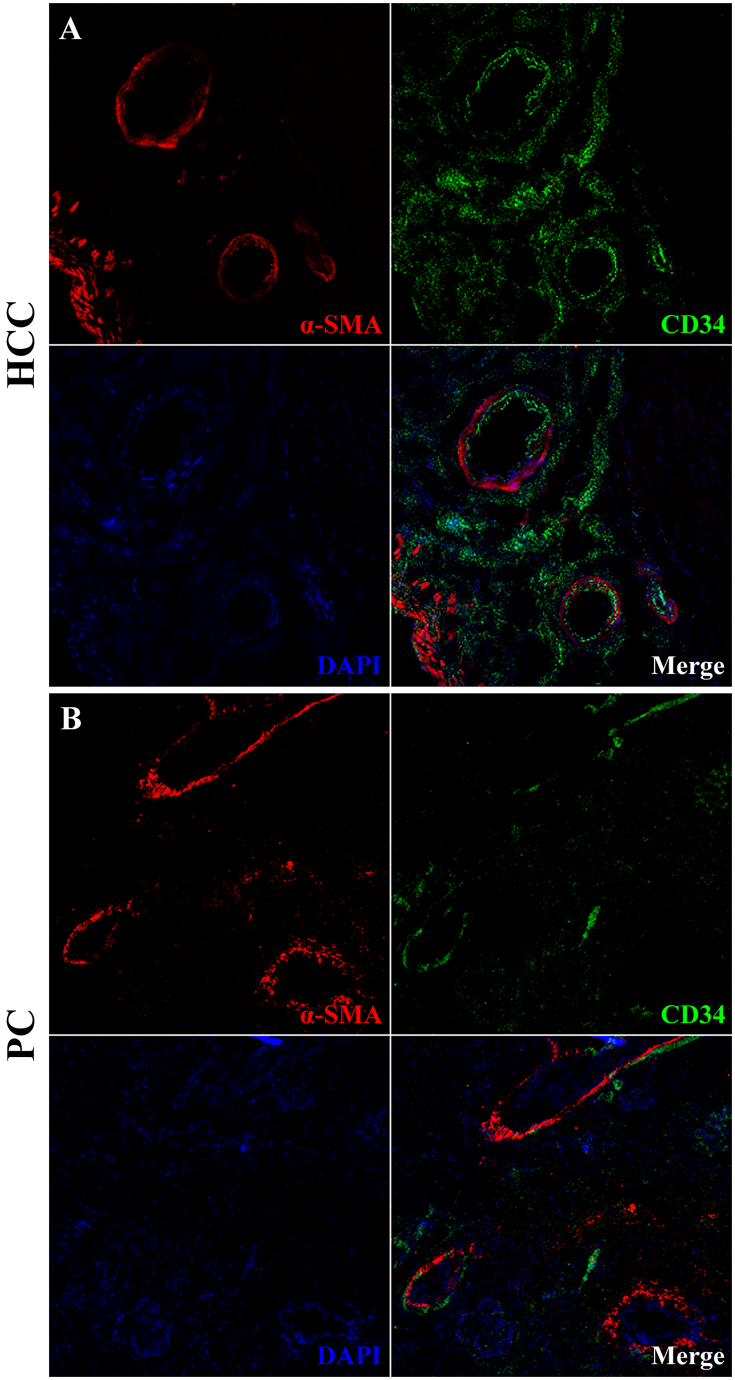


**Figure S4.** Co-distribution of perivascular stromal cells and endothelial cells on tumor vascular wall by immunofluorescent double staining for alpha-smooth muscle actin (α-SMA) and CD34 in (A) hepatocellular carcinoma (HCC) and (B) pancreatic cancer (PC) (laser confocal microscopy, ×250).


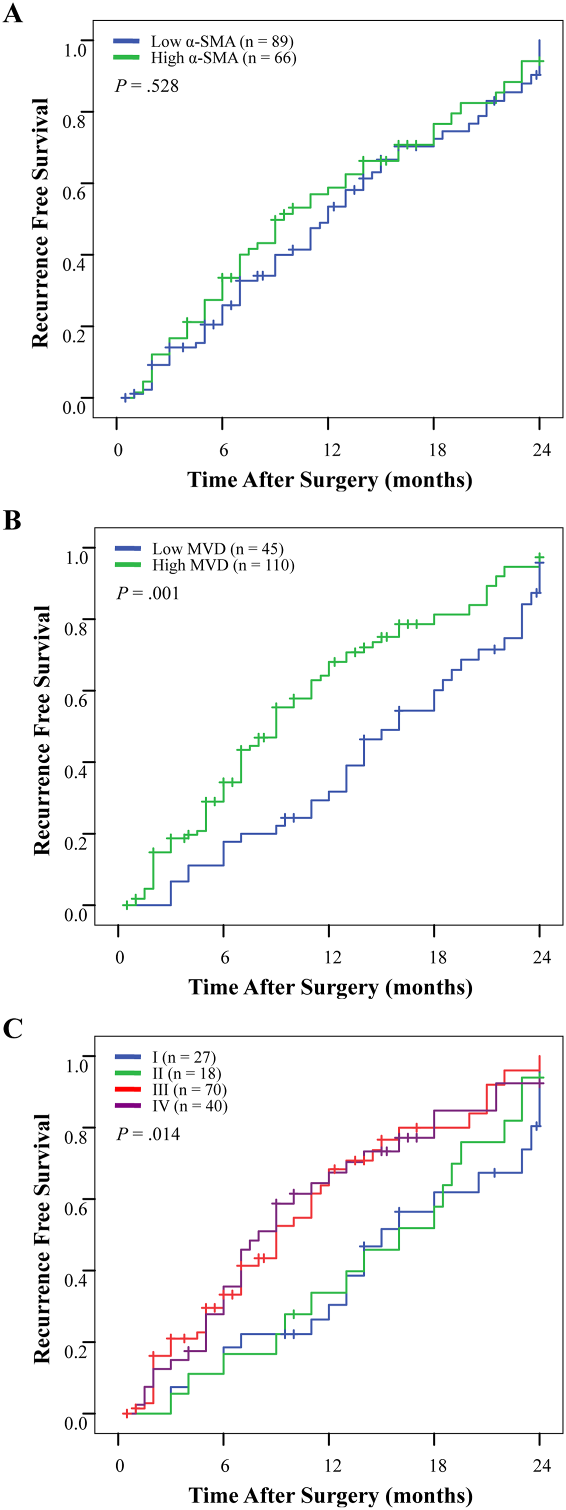


**Figure S5.** Cumulative recurrence-free survival curves of patients with low or high tumor (A) alpha-smooth muscle actin (α-SMA) density or (B) microvessel density (MVD) and (C) their combination in the 2-year recurrence subgroup of hepatocellular carcinoma (see Results for details). Figures were not shown for the late recurrence subgroup.


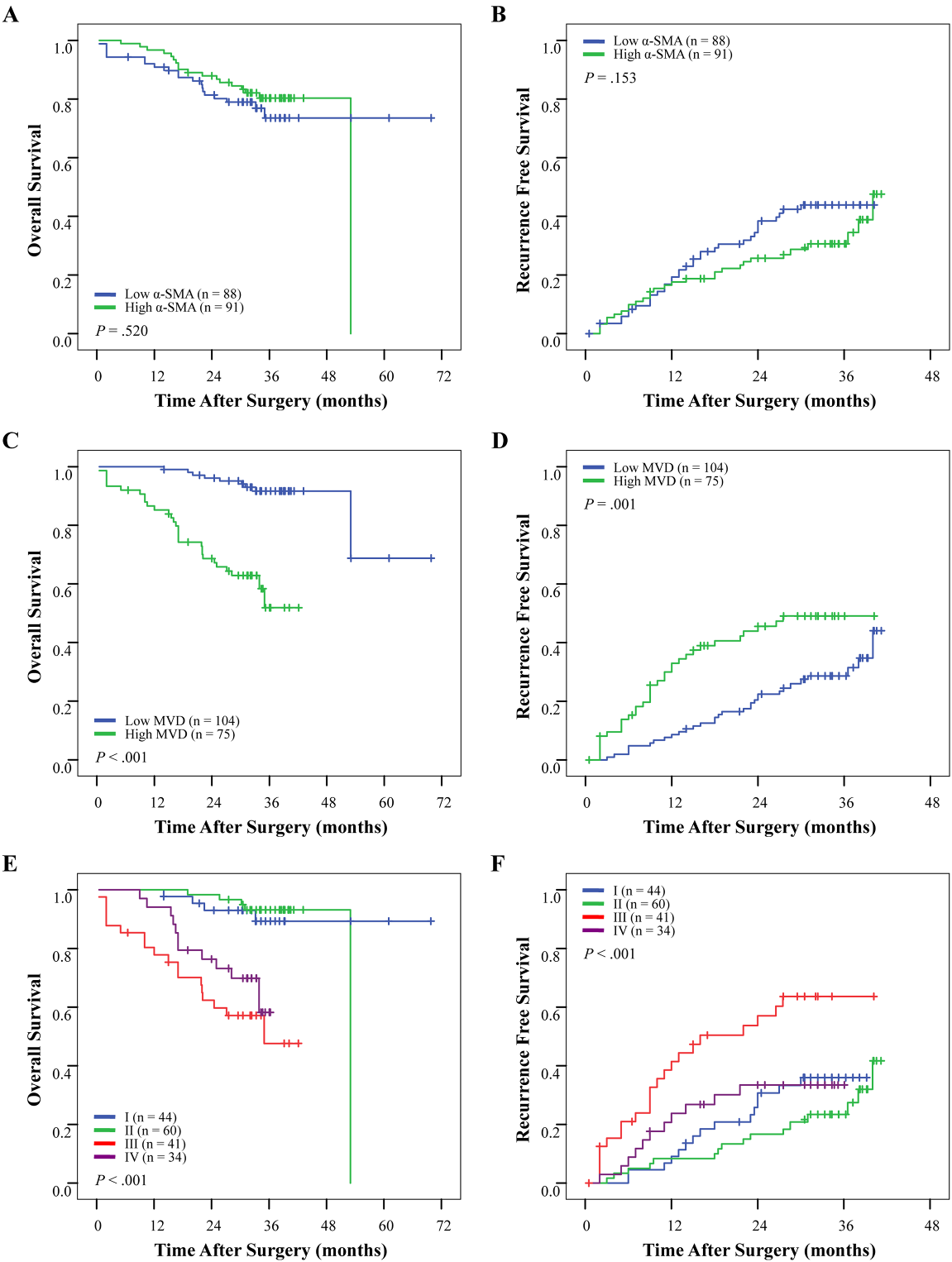


**Figure S6.** Cumulative overall survival (OS) and recurrence-free survival (RFS) curves of patients with low or high tumor alpha-smooth muscle actin (α-SMA) density or microvessel density (MVD) and their combination in the small hepatocellular carcinoma (HCC) subgroup (maximum diameter of ≤ 5 cm; *n* = 179). (A, B) The α-SMA density was associated with neither OS nor RFS. (C, D) Low MVD was associated with prolonged OS and RFS. (E, F) Patients were classified into four groups according to the combination of α-SMA density and MVD. Group II had the best OS and RFS, while group III had the worst OS and RFS.


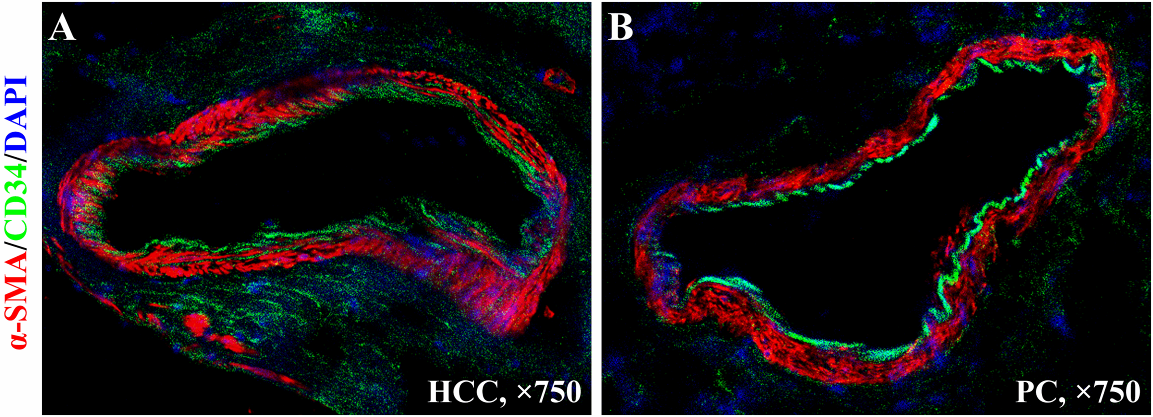


**Figure S7.** Schematic diagram of distribution characteristics of alpha-smooth muscle actin (α-SMA)-positive stromal cells and CD34 in tumor tissue. (Merged A and B) Immunofluorescent double staining of α-SMA and CD34 in frozen sections, showing an α-SMA^+^ (red) cell wrapping around a CD34^+^ (green) cell on the vascular wall (×750).


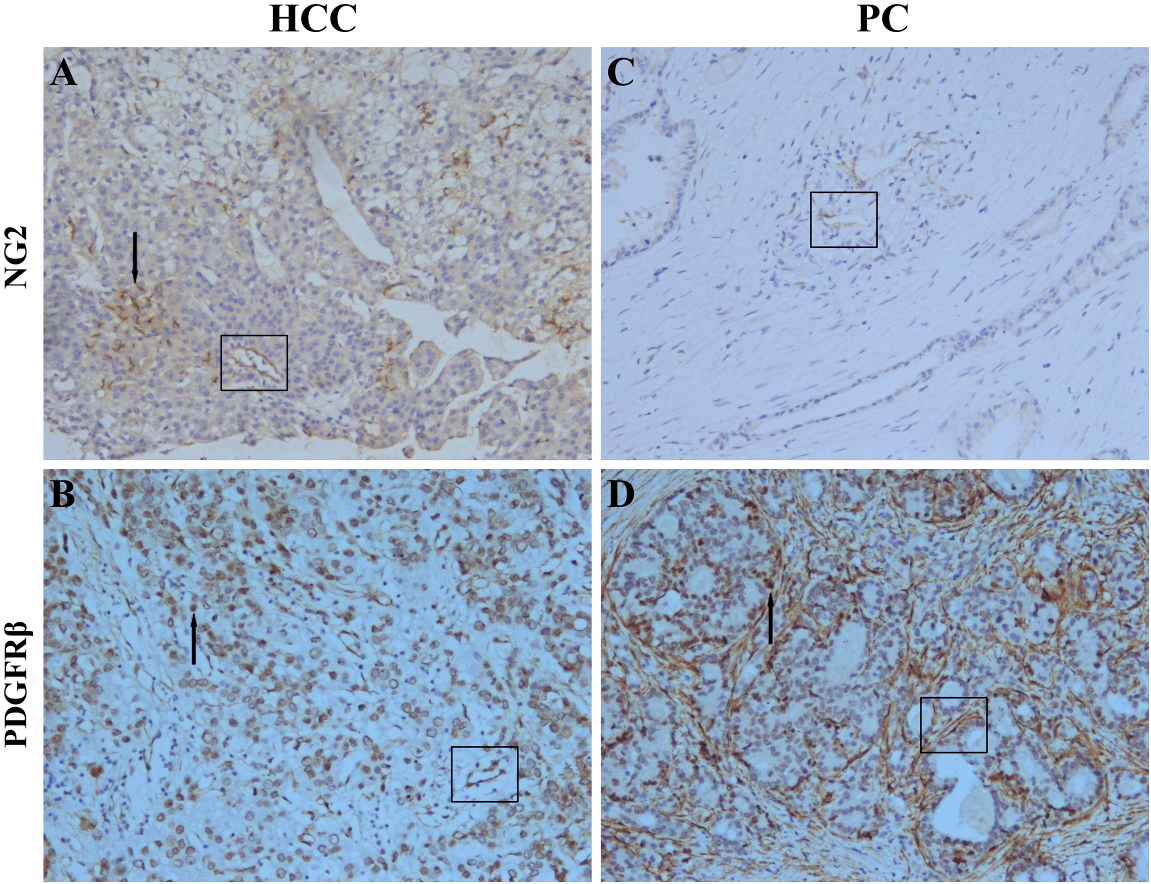


**Figure S8.** Schematic expression of NG2 and PDGFRβ in hepatocellular carcinoma (HCC) and pancreatic cancer (PC). Rectangle shows the typical location of markers in perivascular cells (PVCs). Unfortunately, (A) NG2 staining reveals non-PVC-specific expression in HCC; and (C) almost negative expression in PC (as indicated by arrow). These results were obtained with four different antibodies (Millipore, R&D, Abcam, and Santa Cruz), all employing the same immunohistochemistry protocol. (B, D) PDGFRβ staining also showed positive expression in tumor cell nuclei (as indicated by arrow); hence, it could not be used as a marker in quantitative analysis of PVCs. Moreover, neither NG2 nor PDGFRβ was found to be associated with patient outcome, either separately or in cooperation with MVD.
